# Age- and sex-specific percentile curves for gross and fine motor skills in early childhood: an analysis from the SUNRISE International Study

**DOI:** 10.1007/s00431-026-07249-y

**Published:** 2026-07-21

**Authors:** Clarice Martins, E. Kipling Webster, Vicente Romo-Perez, Ankhmaa Byambaa, Cain Clark, Chalchisa Abdeta, Dyah Anantalia Widyastari, Denise Lian, Eva Roos, Elina Engberg, Kar Hau Chong, Piyawat Katewongsa, Tawonga Mwase-Vuma, Anthony Okely

**Affiliations:** 1 Research Centre in Physical Activity, Health and Leisure, Laboratory for Integrative and Translational Research in Population Health, University of Porto, Porto, Portugal; 2 Department of Kinesiology, Recreation, and Sport Studies, University of Tennessee, Knoxville, TN USA; 3 CIDEGA, Faculty of Education and Sports Sciences, Universidade de Vigo, Vigo, Spain; 4 School of Medical, Indigenous and Health Sciences, Faculty of Science, Medicine and Health, University of Wollongong, Wollongong, NSW Australia; 5 Faculty of Health, Education, and Life Sciences, City South Campus, Birmingham City University, Westbourne Road, Edgbaston, UK; 6 School of Education, University of Wollongong, Wollongong, Australia; 7 Institute for Population and Social Research, Mahidol University, Salaya, Phutthamonthon, Nakhon Pathom Thailand; 8 Thailand Physical Activity Knowledge Development Centre, Salaya, Phutthamonthon, Nakhon Pathom Thailand; 9 Centre for Education & Community Well-Being, Faculty of Education, University Kebangsaan, Bandar, Baru Bangi, Malaysia; 10 Folkhälsan Research Center, Helsinki, Finland; 11 Faculty of Medicine, University of Helsinki, Helsinki, Finland; 12 School of Education, Early Start, Faculty of Arts, Society and Businesss, University of Wollongong, Wollongong, NSW Australia; 13 Department of Psychological Sciences and Health, University of Strathclyde, Glasgow, Scotland; 14 Centre for Social Research, University of Malawi, Zomba, Malawi

**Keywords:** Motor development, Percentile curves, Fine motor skills, Gross motor skills, Preschoolers

## Abstract

Motor skills during early childhood are shaped by biological maturation, environmental opportunities, and cultural contexts. However, most reference data on motor skills derive from high-income countries, limiting global comparability. The SUNRISE International Study was developed to address this gap by generating cross-cultural evidence on early child development. This study aimed to develop age- and sex-specific percentile curves for gross and fine motor skills, based on a cross-cultural and income-diverse sample of 3–4-year-old children included in the SUNRISE Study. In 6 countries, 3027 children completed the supine-timed up and go and the 9-hole pegboard test, as measures of gross and fine motor skills, respectively. The Generalized Additive Models for Location, Scale and Shape model, with the Box-Cox Power Exponential adjusted method was used to construct percentile curves within each 3-month age category, according to sex. Percentile curves were generated to offer a foundational reference for interpreting gross and fine motor skills development within the scope of the SUNRISE. The proposed curves may support tracking children’s motor skills and inform context-specific strategies aimed at identifying and addressing potential motor skills delays, while maintaining comparability across countries using the harmonized protocols. Regular updates based on broader and representative datasets are encouraged to maintain their relevance and applicability across populations and countries.

**Table:** 

**What is Known:** • *Motor competence (MC) assessment is essential for identifying developmental delays and guiding interventions in early childhood.* • *Existing reference values for gross and fine MC often have limited applicability across diverse populations due to cultural and contextual differences.*	
**What is New:** • *The percentile curves developed provide a robust framework for comparing gross and fine MC across different samples within the scope of the Sunrise International Study.* • *These curves, capturing a broader range of variability, offer guidance for comparing children´s motor development, supporting teachers, health professionals, and policy makers at promoting motor skill development.*

**Supplementary Information:**

The online version contains supplementary material available at 10.1007/s00431-026-07249-y.

## Introduction

Early childhood experiences work alongside genetic variations and epigenetic regulation to generate positive developmental trajectories, impacting health across the life course [1]. The early childhood years are a critical time for motor development [2], a fundamental component for a healthy lifestyle [3] and lifelong health.

Motor development refers to the progression of physical abilities that involve the movement and coordination of muscles [4] and encompasses both gross and fine motor skills. Gross motor skills refer to movements that require the use of large muscle groups, involving large extremities and often the entire body, while fine motor skills are commonly thought of as small muscle movements requiring close eye–hand coordination (e.g., writing or tying a shoe) [5]. Both domains of motor skills are dependent on the condition of the child’s neuromuscular and neurodevelopmental status, and the quality of the child's movement patterns [6], to produce embodied movements, embedded actions, and enculturated interactions [7].

Children´s motor skills development is the product of growth and maturation, environmental affordances, and cultural interactions. Understanding of children’s motor development is largely based on research from high-income countries. Little is known among children from low- and middle-income countries (LMICs) [8], some of which are undergoing rapid urbanization, which may limit children´s movement possibilities (i.e., less access to outdoor free play, fewer opportunities for active commuting), and negatively impacting their motor development.

The SUNRISE International Study (https://sunrise-study.com) was designed to fulfill this knowledge-to-action gap of data from LMICs, determining the proportion of 3- and 4-year-old children who meet the WHO Global guidelines [9] for physical activity, sedentary and sleep behaviors, and associating these movement behaviors with health and development outcomes, such as overweight and obesity, executive function, and gross and fine motor skills. The SUNRISE International Study aims to reflect the context, the content, and the cultural background of children´s development and is the largest-known ongoing global research project for the early years, comprising 71 countries to date. In terms of motor skill performance, the SUNRISE protocol [10] assesses both gross and fine motor skills, based on tests that are understandable for practitioners and stakeholders of diverse ethnic origin, and are easy to undertake, score, and interpret, with minimal cost.

To assess children’s gross motor skills, the SUNRISE protocol uses a measure of locomotion and mobility through the Supine-Timed Up and Go (STuG) test [10]. For fine motor skills, the 9-hole pegboard test is used to assess manual dexterity, which is a central component of hand function, related to both the speed and accuracy of hand movements [11]. It is related to an individual’s ability to coordinate the fingers and manipulate objects in a timely manner [12]. Such ability impacts the individual’s performance in daily activities (bathing, grooming, eating), completing work related tasks, and engaging in leisure activities. It is also reported as an indicator of academic achievement in childhood, such as handwriting [13].

The absence of age-specific percentile curves against which young children’s scores can be compared is a challenge for researchers and practitioners to accurately report how the children assessed in the SUNRISE International Study performed on the STuG and the 9-hole pegboard tests. Previous research has reported evidence of validity for the 9-hole pegboard test [12] and described reference values across the age span (3–85 years) for a representative sample of US population-data [14]. Nonetheless, the curves for the early childhood were solely based on data of 331 preschool children aged 3–4 years from the USA, reflecting a demographic that does not accurately represent manual dexterity developmental profiles in other social and cultural contexts from where data was not provided. Concerning children´s gross motor skills, to the best of the authors’ knowledge, there is no available percentile values for the STuG. Consequently, this hinders analyzing combined data from various countries using uniform age-specific parameters.

Children’s competency to perform motor skills is shaped not only by biological maturation, but also by environmental affordances, educational practices, opportunities for active play, socioeconomic conditions, and cultural expectations surrounding children’s movement behaviors. Consequently, reference values derived exclusively from single-country or high-income settings may not adequately reflect the diversity of developmental trajectories observed globally. The establishment of percentile curves for the STuG and the 9-hole pegboard test using pooled data from diverse cultural contexts may contribute to a broader understanding of the differences in motor development patterns among children from geographically, culturally, and economically diverse countries. By providing a more ecological characterization of early childhood motor skills, these percentile curves may serve as benchmarks for assessing children’s performance, identifying periods for skills acquisition or stability, while supporting comparability through harmonized assessment protocols. This information may also help support the design of interventions aimed at promoting physical activity and addressing delays in motor development in children worldwide.

Therefore, the current study aimed to develop age- and sex-specific percentile curves for the STuG and the 9-hole pegboard test, based on a cross-cultural and income-diverse sample of 3–4-year-old children included in the SUNRISE International Study. The results presented provide the typical developmental trajectories of children’s gross and fine motor skills from a large-scale dataset and offer reference points against which children’s performance can be compared.

## Methods

The Strengthening the Reporting of Observational Studies in Epidemiology (STROBE) guidelines for cross-sectional studies were adhered to for the current study [15].

### Data sources and participants

SUNRISE is an international cross-sectional surveillance study examining 24-h movement behaviors and developmental/health outcomes in the early years of life across geographically, culturally, and economically diverse countries [10] using harmonized protocols and targeting approximately 50% of children from rural and urban areas. To be eligible for the study, children needed to be between the ages of 3.0 and 4.9 years old, with no parent-reported mobility limitations.

Within the SUNRISE International Study protocol, the only exclusion criterion was physical limitation related to engaging in ambulatory behaviors. Children were not excluded based on neurodevelopmental disorders, cognitive impairments, or other clinical conditions. In several countries, formal diagnoses are often not yet completely established at these ages. Children were considered eligible if they were able to understand, participate in, and complete the assessment procedures. This approach was intentionally adopted to maximize inclusiveness and ecological validity, reflecting the diversity typically observed in real-world early childhood populations across participating countries. Therefore, the exclusion criteria in the present study were restricted to incomplete or missing data required for the specific analyses conducted.

Due to financial or logistic constraints, data collection does not occur simultaneously across all participating countries. At the time the present analyses were conducted, complete datasets were available from six participating countries that had finalized data collection, processing, and quality control procedures according to the SUNRISE protocol. Data reported in this paper represent a large multicenter collaboration comprising data from the SUNRISE main studies conducted in Ethiopia, Finland, Malawi, Mongolia, Papua New Guinea, and Thailand. The analyses were conducted using a complete-case approach, including only children with complete measures for all variables of interest; participants with missing data for any outcome were excluded, and no imputation procedures were applied.

In Ethiopia, children were recruited in 18 kindergartens in Adama city and 11 rural villages in Lume districts, East Shewa Zone, Oromia regional state from 1 April 2022 to 30 September 2022. In rural villages, there are no kindergartens. Community health extension workers provided a list of eligible children from their health registry, and data collectors approached parents in person.

In Finland, data assessments were done in children from the cities of Helsinki, Turku, Kuopio, Oulu, Espoo, and the surrounding rural areas of each city (57% in urban and 43% in rural areas) [16]. They were assessed between June 2022 and November 2023 in 177 early childhood education and care (ECEC) centers.

In Malawi, children were recruited from 20 ECEC centers in urban (*n* = 6) and rural (*n* = 14) areas of Zomba district, Southern Malawi, between August 2022 and May 2023. ECEC centers located within reasonable travel distance from Zomba City Centre (where the Country Chief Investigator’s institution was based) were selected from a list of centers maintained by the local government’s social welfare offices in the district. The Malawian sample oversampled rural children (76%) to reflect the country’s sociodemographic and economic variations [17] (i.e., most people live in rural settings and are poor).

Mongolian children were recruited using convenience sampling from three kindergartens in Ulaanbaatar city and 2 kindergartens in Tuv province. The Ministry of Education and Science’s Department of Preschool Education assisted in identifying kindergartens for the data collection. Ulaanbaatar city does not have rural areas by the official definition. Thus, one kindergarten located on the city’s outskirts in the district of Dambadarjaa (Sukhbaatar district) was recruited as a proxy for rural setting in Ulaanbaatar city. In Mongolia, kindergartens were attended by children aged 2–5 years. For the purpose of this study, children aged 3 and 4 years who were assessed were included in the sample. Data collection occurred between December 2021 and April 2022.

In Papua New Guinea, data collection occurred from January to December 2020 and was carried out in the communities. Potential child participants were identified from the household socioeconomic surveillance database. Prior to the data collection, the national surveillance team was trained about the overall objective and methods of the study. Parents and caregivers of children who lived in the surveillance sites were informed about the study and provided written informed consent. The gross and fine motor skill tests were conducted by trained interviewers during their visits to households/villages. The testing processes were explained to parents/caregivers as well as the child participants in their local languages.

In Thailand, children were recruited from 33 ECEC centers across the country. The sample was selected using multistage random sampling from a nationally representative group of children aged 3 to 4 years. Data collection took place between January and March 2023, of which face-to-face interviews with parents and assessments of the children were conducted in the ECEC settings.

Ethics committees in each participating country approved the studies, and written informed consent was obtained from all parents/guardians. Representatives from each participating country took permission responsibility for sharing data for the secondary analysis.

### Sample size estimation and justification

Given the SUNRISE study observational and descriptive nature, the sample size was determined to prioritize feasibility, global diversity, and cross-country comparability, rather than formal power calculations for hypothesis testing. A recommended target of 1000 preschool-aged children per country stratified by age group and urban/rural residence was established. To date, only a limited number of countries have achieved this target. Accordingly, the present study includes all available data from participating low-, middle-, and high-income countries. Recruitment in each country was based on convenience sampling and was not intended to generate nationally representative country-level estimates.

Appropriate statistical methods were applied to ensure the reliability of the resulting curves, acknowledging the non-linear and non-representative nature of the sample.

### Measurements

All measurements were collected following a standardized protocol, which included an initial data collectors training by the same instructor in all countries. These trained data collectors and research staff subsequently conducted assessments with children in ECEC centers.

#### Motor skills

The STuG and the 9-hole pegboard test were motor skills protocols selected as proxy indicators of gross and fine motor skills primarily due to their feasibility within the context of large-scale international surveillance. These assessments require little or no specialized equipment, involve relatively low cost, require minimal administration time, do not require large physical spaces, and can be administered with limited training or expertise. Such characteristics support their applicability across diverse contexts and facilitate implementation in large population-based studies. Importantly, the feasibility and acceptability of these assessments were previously evaluated in pilot studies across participating SUNRISE countries before their inclusion in the protocol.

#### Gross motor skills

The STuG is recommended by the Motor Domain Group for the National Children’s Study (USA) [10], as an assessment for general mobility and locomotion. A line was marked 3 m from a wall (using tape or chalk), and a target was marked on the wall at the child’s eye level. The child lay supine on their back with their heels on the line. On “go,” the child was required to get up as quickly as possible, run, touch a target fixed on the wall, and run back across the 3 m line. Timing was started when the assessor said “Go” and stopped as soon as the child’s torso crossed the line. This task consisted of one practice trial and two test trials, with the child’s fastest time in seconds of the two test trials used for analyses.

#### Fine motor skills

The 9-hole pegboard test (PAT-A8515, Sammons Preston, Illinois, USA) was based on the assessments in the NIH Toolbox Motor Battery (ages 3–6) [11]. This battery of assessments is included as a key domain in the NIH Toolbox for Assessment of Neurological and Behavioral Function. Its subdomains measure dexterity, strength, balance, locomotion, and endurance. This battery has demonstrated excellent test–retest reliability with all measures meeting ICC > 0.80, and demonstrating criterion validity of r > 0.75 [11]. The 9-hole pegboard test was used to assess manual dexterity. The child was timed while picking up nine pegs with the right hand, one at a time, and inserted them into the pegboard (31.1 cm × 26.0 cm × 4.3 cm). Once all pegs were placed in the holes, they were asked to take them back out and place in the bowl. The test was repeated with the left hand. The timer began as soon as the assessor said “Go” and was stopped as soon as the final peg was placed back in the original position (well) in the pegboard.

Although children may begin to show preference for the right or left hand when they are 2–3 years old [14], or even earlier, and the developmental milestone for handedness is about 24 months, a great variability is observed between children [18]. After inspection of the data, and based on the lack of criteria to define preferred and non-preferred hands, data were operationalized as right and left hands, as well as the sum of both hands, following the standardized protocol to collect data in countries that comprise the SUNRISE International Study.

### Statistical analysis

Data from countries were pooled into a single dataset to increase statistical power and to allow for generalizable modelling across the full sample. No imputation of information was performed; only complete cases were included in the analyses, representing 96% of the total sample of the six countries. Results for gross and fine motor skills were inspected for the normality of distribution according to age (3-month interval) in months and sex. To ensure comparable age distributions between sexes, a *t*-test was used to assess differences in age between boys and girls. Participants were stratified by sex within each 3-month age category for subsequent modeling. Age ranged from 36 to 59 months; however, because age groups were organized in 3-month intervals, the final interval is presented with an upper boundary of 60 months. Developmental centile curves were estimated using Generalized Additive Models for Location, Scale, and Shape (GAMLSS), employing the Box-Cox Power Exponential (BCPE) distribution, which allows flexible modeling of non-normal distributions. This method was selected due to its ability to flexibly accommodate the skewed and kurtotic distributions commonly observed in childhood motor development. Indeed, the BCPE distribution models four parameters: *µ* (location; central tendency), *σ* (scale; dispersion), *ν* (skewness), and *τ* (kurtosis), thus offering superior performance over traditional normal-based models for capturing growth and motor performance patterns [19]. This method has been extensively validated and is used in constructing growth standards, including the WHO child growth references [20]. Cubic spline smoothing functions were applied to the *µ* and *σ* parameters as functions of age (in months), permitting the modelling of non-linear developmental trends. The optimal degrees of freedom for each spline were estimated automatically using the Generalized Akaike Information Criterion (GAIC), which balances model fit against complexity and reduces the risk of overfitting. Moreover, model adequacy was assessed through a combination of diagnostic tools, including quantile residual plots, density plots, Q–Q plots, and Filliben correlation coefficients, to evaluate residual normality and overall model fit [21]. These diagnostics confirmed acceptable model performance across all age-sex strata.

Given known sex differences in motor development, separate models were developed for boys and girls in relation to gross and fine motor skills. Due to limited variability in STuG scores, percentile estimates were focused on the 10th to 90th range to capture central trends; specifically, the 10th, 25th, 50th, 75th, and 90th percentiles were established. For the 9-hole pegboard test, the 3rd, 10th, 25th, 50th, 75th, 90th, and 97th percentiles were estimated, to allow for higher sensitivity and consideration of a wider spread of scores. All analyses were performed in R (R Core Team (2023). R: A Language and environment for statistical computing. [Computer software; retrieved from https://cran.r-project.org/), using the GAMLSS (v5.4.22) package [22].

## Results

**Fig. 1:**
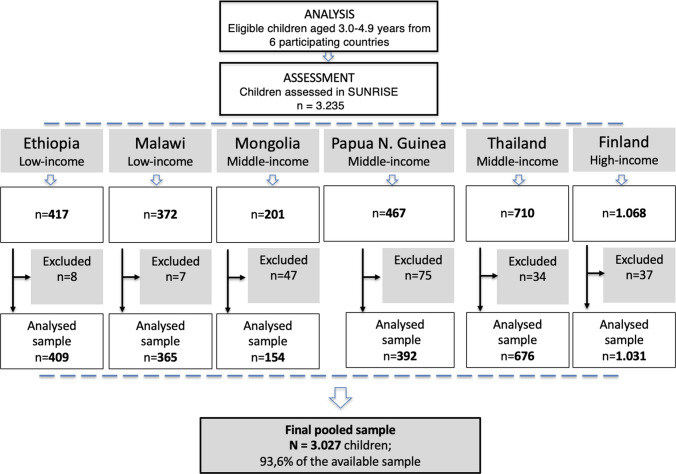
Descriptive of study participants within country and country income level

The final dataset included 3027 children who completed the entire protocol. A balanced distribution between boys and girls (50.8% boys) was achieved. The distribution of study participants within country and country income level is presented in Fig. 1.

Table 1 presents motor skill performances according to country income level and sex. For the 9-hole pegboard test with the right hand, boys differed significantly across all three income groups, with the fastest performance observed in middle-income countries and the slowest performance in high-income countries. Among girls, significant differences were observed between low- and middle-income groups and between middle- and high-income groups, with girls from middle-income countries showing the fastest performance. For the left hand, significant differences were also observed across income groups. In both boys and girls, post hoc comparisons indicated significant differences between low- and middle-income countries and between middle- and high-income countries. Overall, children from middle-income countries showed faster performance than those from low- and high-income countries.

**Table 1:** Sex-specific differences in 9-hole pegboard and STuG performance across country income groups

Variable	Ethiopia, Malawi (*n* = 774)		Mongolia, Papua N. Guinea, Thailand (*n* = 1.222)		Finland (*n* = 1.031)	
	Low income		Middle income		High income	
	Boys	Girls	Boys	Girls	Boys	Girls
Right hand (sec)	41.8 ± 10.4	39.3 ± 10.4^d^	39.5 ± 10.7^a^	36.9 ± 10.3^a,b,d^	44.0 ± 12.6^b^	39.7 ± 10.5^d^
Left hand (sec)	48.0 ± 12.8	47.4 ± 12.5	44.3 ± 11.9^a^	42.4 ± 11.7^a,b,d^	47.7 ± 13.4^b^	45.8 ± 12.6^d^
STuG (sec	7.3 ± 3.0	7.3 ± 3.1	7.00 ± 3.5	6.8 ± 2.7^a^	7.1 ± 3.4	6.8 ± 2.4

sec, seconds; right hand, pegboard score for the right hand; left hand, pegboard score for the left hand

^a^Differences between low vs middle income

^b^Differences between middle vs high income

^c^Differences between low vs high

^d^Differences between boys vs girls

For the STuG test, no statistically significant differences across income groups were observed among boys. Among girls, a significant difference was found only between low- and middle-income countries, with girls from middle-income countries showing slightly faster performance. No other significant differences were observed for STuG.

The mean score and dispersion values in gross and fine motor skills (STuG and pegboard, respectively), stratified by age in months, and sex are reported in Table 2.

**Table 2:** Descriptive of scores for gross and fine motor skills stratified by age in 3-month period and sex

Age (months)	STuG			Pegboard right			Pegboard left		
	Boys	Girls	Total	Boys	Girls	Total	Boys	Girls	Total
36	8.7 ± 1.8	9.1 ± 2.7	8.9 ± 2.3	52.4 ± 13.1	51.6 ± 15.2	52.0 ± 14.0	61.0 ± 13.7	62.3 ± 16.9	61.6 ± 15.2
37	8.7 ± 3.0	9.1 ± 3.0	9.0 ± 3.0	55.4 ± 13.7	48.5 ± 11.2	51.6 ± 12.8	60.2 ± 15.9	56.3 ± 12.7	58.1 ± 14.3
38	7.7 ± 2.0	7.6 ± 2.0	7.6 ± 2.0	50.5 ± 12.1	45.0 ± 10.6	47.7 ± 11.6	55.2 ± 16.3	53.5 ± 11.8	54.4 ± 14.1
39	7.4 ± 1.9	8.2 ± 2.2	7.8 ± 2.2	50.8 ± 10.7	43.9 ± 91	47.6 ± 10.5	55.5 ± 12.7	53.313.2	54.4 ± 12.9
40	7.82.3	8.2 ± 2.5	8.0 ± 2.5	48.4 ± 10.1	44.4 ± 8.5	46.5 ± 9.5	55.6 ± 15.4	51.0 ± 9.5	53.4 ± 13.0
41	7.7 ± 2.7	7.8 ± 2.3	7.8 ± 2.3	46.5 ± 8.7	43.5 ± 11.3	45.1 ± 10.1	53.3 ± 11.6	53.1 ± 12.7	53.2 ± 12.1
42	8.4 ± 3.2	7.6 ± 2.7	7.9 ± 2.7	45.3 ± 12.6	43.2 ± 12.1	44.1 ± 12.4	51.6 ± 12.3	47.9 ± 10.8	49.6 ± 11.6
43	7.4 ± 2.6	6.9 ± 2.1	7.1 ± 2.1	44.2 ± 9.5	42.0 ± 9.2	42.9 ± 9.4	49.9 ± 10.6	47.9 ± 12.4	48.7 ± 11.7
44	8.2 ± 2.8	7.4 ± 2.6	7.8 ± 2.6	46.5 ± 11.5	41.5 ± 9.3	44.2 ± 10.8	53.4 ± 14.3	47.8 ± 11.2	50.8 ± 13.2
45	7.4 ± 2.3	7.2 ± 2.0	7.3 ± 2.0	43.4 ± 10.3	39.4 ± 9.5	41.5 ± 10.1	47.8 ± 12.4	46.5 ± 13.3	47.2 ± 12.8
46	7.4 ± 3.0	6.9 ± 2.5	7.1 ± 2.5	45.7 ± 12.2	40.1 ± 9.4	42.8 ± 11.2	50.1 ± 11.0	47.8 ± 13.6	49.0 ± 12.4
47	7.1 ± 1.9	6.9 ± 1.8	7.0 ± 1.8	44.0 ± 11.3	40.2 ± 9.5	41.9 ± 10.5	46.9 ± 9.6	46.3 ± 10.3	46.6 ± 10.0
48	6.8 ± 1.8	7.1 ± 2.3	6.9 ± 2.3	40.0 ± 10.1	36.0 ± 8.5	38.2 ± 9.6	44.2 ± 10.3	43.6 ± 11.7	43.9 ± 10.9
49	6.4 ± 1.6	7.0 ± 1.8	6.7 ± 1.7	41.3 ± 11.4	37.1 ± 6.9	39.4 ± 9.9	46.5 ± 11.8	42.6 ± 9.7	44.8 ± 11.1
50	6.6 ± 1.6	7.0 ± 2.4	6.8 ± 2.0	41.3 ± 11.0	36.5 ± 8.2	39.1 ± 10.1	46.4 ± 11.7	43.6 ± 10.8	45.2 ± 11.3
51	6.5 ± 1.7	6.3 ± 1.5	6.4 ± 1.6	38.0 ± 8.8	36.0 ± 8.7	37.0 ± 8.8	42.6 ± 10.2	40.8 ± 8.9	41.7 ± 9.6
52	6.2 ± 1.8	6.2 ± 1.6	6.2 ± 1.7	36.2 ± 7.9	35.6 ± 10.0	35.9 ± 8.9	40.4 ± 9.1	41.2 ± 8.9	40.8 ± 9.0
53	6.4 ± 1.8	6.8 ± 2.4	6.6 ± 2.1	37.5 ± 8.7	34.2 ± 9.9	35.8 ± 9.4	43.0 ± 9.1	40.1 ± 11.2	41.5 ± 10.3
54	6.2 ± 2.1	5.8 ± 1.4	6.0 ± 1.8	36.5 ± 8.5	34.2 ± 8.1	35.5 ± 8.4	40.9 ± 9.5	39.5 ± 9.0	40.3 ± 9.3
55	6.2 ± 1.7	6.0 ± 1.5	6.1 ± 1.6	36.6 ± 8.2	33.5 ± 6.2	34.8 ± 7.3	42.2 ± 10.0	38.9 ± 9.1	40.3 ± 9.6
56	5.8 ± 1.1	6.1 ± 1.3	5.9 ± 1.2	34.7 ± 6.3	31.9 ± 7.4	33.4 ± 7.0	38.0 ± 6.7	38.8 ± 11.4	38.4 ± 9.3
57	5.8 ± 1.4	6.2 ± 1.9	6.0 ± 1.7	35.3 ± 10.2	32.3 ± 5.7	33.6 ± 8.1	37.0 ± 8.6	37.7 ± 7.0	37.4 ± 7.7
58	6.0 ± 1.5	6.1 ± 1.8	6.0 ± 1.7	35.8 ± 10.1	32.3 ± 8.1	34.4 ± 9.5	39.0 ± 9.2	38.0 ± 8.6	38.6 ± 8.9
59	5.9 ± 1.7	6.0 ± 1.5	5.9 ± 1.6	33.9 ± 7.2	33.1 ± 6.3	33.5 ± 6.8	37.3 ± 7.3	38.9 ± 8.6	38.1 ± 7.9
Total	6.9 ± 2.2	6.9 ± 2.1	6.9 ± 2.2	41.7 ± 11.5	38.4 ± 10.3	40.1 ± 11.1	46.4 ± 12.8	44.9 ± 12.4	45.7 ± 12.7

Scores are expressed in seconds

**Fig. 2:**
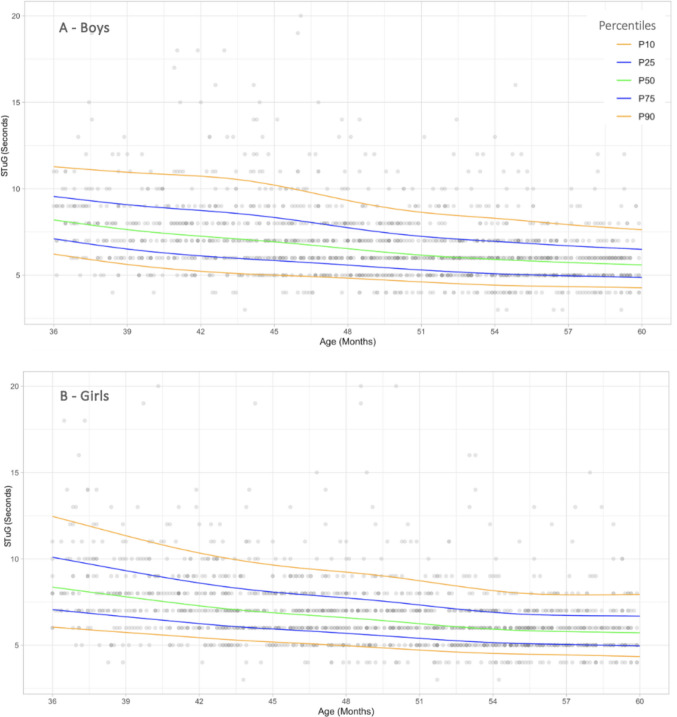
Gross motor skills percentile curves based on the STuG for boys (**A**) and girls (**B**)

In general, high variability in gross motor skills according to age and sex was observed (Fig. 2A and B). The STuG percentile curves depicted quite similar performances for boys (Panel A) and girls (Panel B) up to the 50th percentile, whereafter boys’ curves showed similar or slightly better performance than girls. Between the 75th and the 90th percentiles, a greater range between rank curves was seen for both boys and girls. In general, StuG performance showed decreasing scores across age, indicating shorter completion time and therefore, better motor performance as children grew older. This trend was slightly less evident among girls, in the last age interval (i.e., 57 to 60 months), where performance appeared to stabilize.

The corresponding percentile tables including the age- and sex-specific distribution parameters (*μ*, *σ*, *ν*, *τ*) for STuG are available as supplementary files (Supplementary file 2—Tables S1 and S2). The diagnostics plots and the selected modelling parameters for the final models are available as supplementary files (Supplementary file 1—Figs. 1 and 2).

**Fig. 3:**
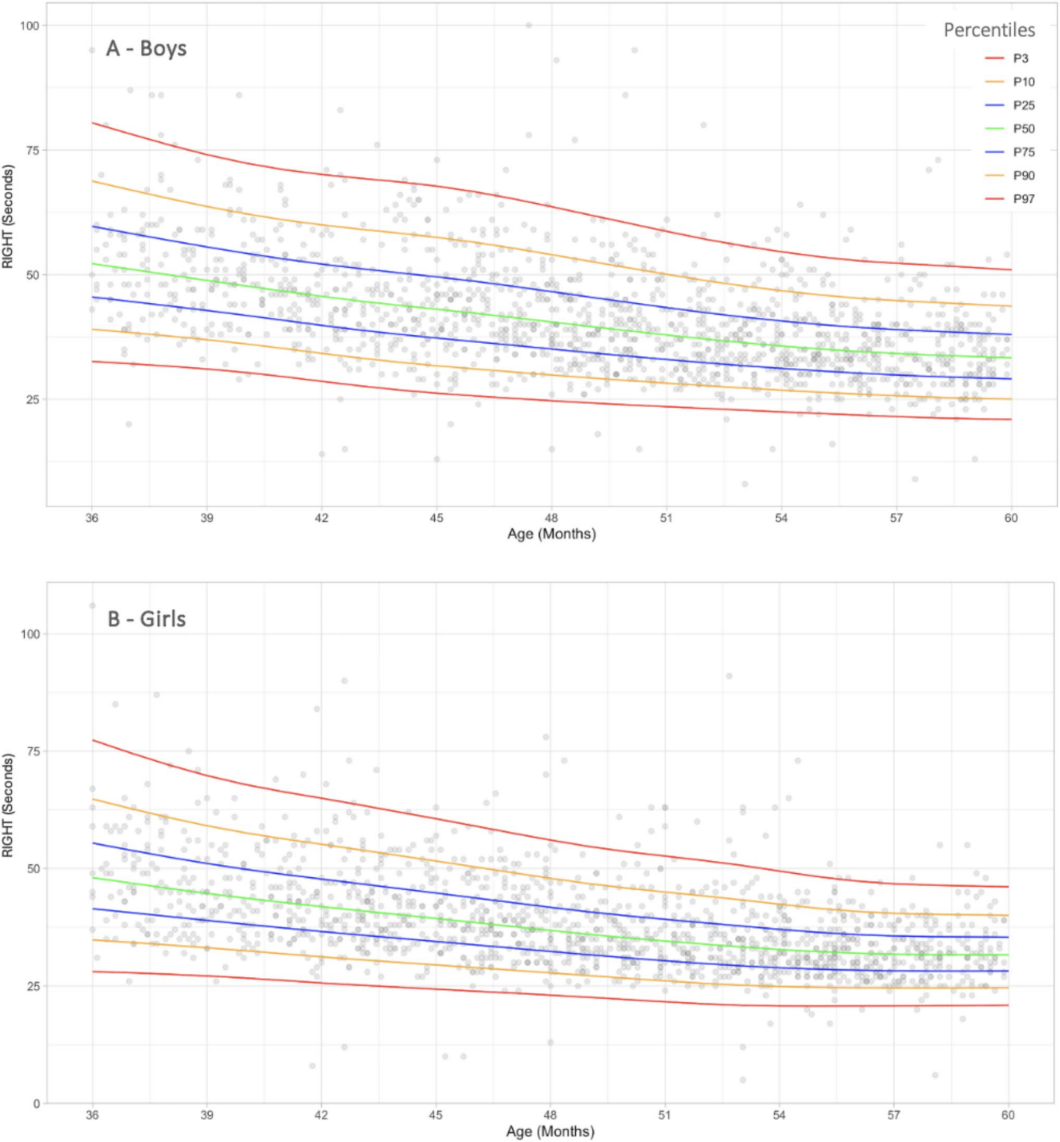
Fine motor skills percentile curves for the right hand based on the 9-hole pegboard test for boys (**A**) and girls (**B**)

**Fig. 4:**
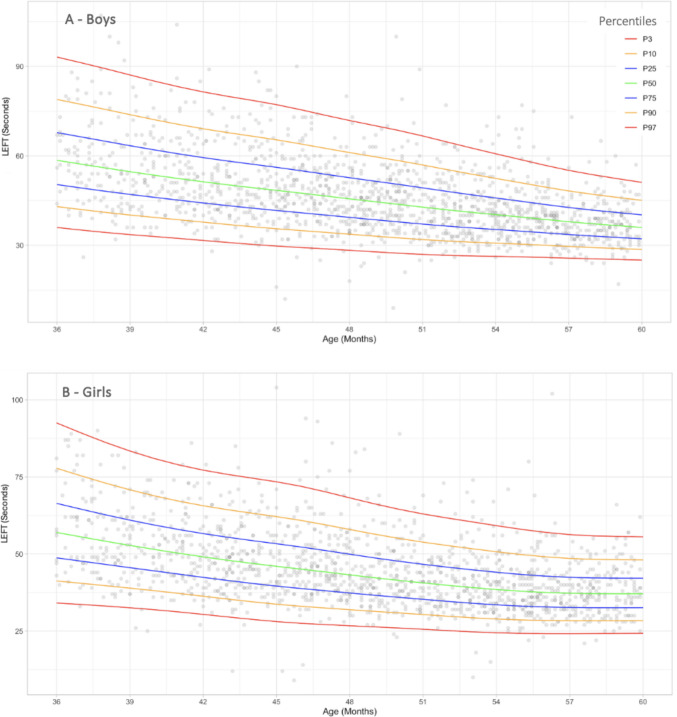
Fine motor skills percentile curves for the left hand based on the 9-hole pegboard test for boys (**A**) and girls (**B**)

**Fig. 5:**
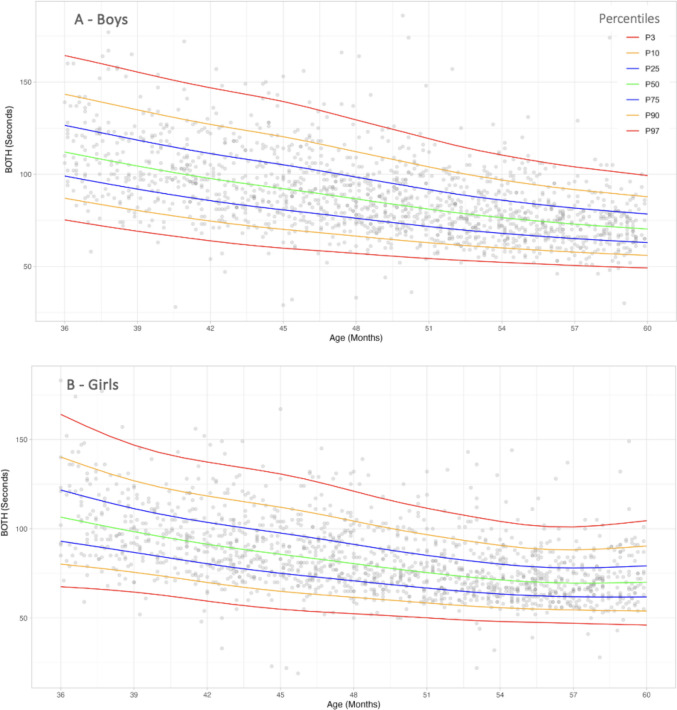
Fine motor skills percentile curves for the sum of the values in both hands based on the 9-hole pegboard test for boys (**A**) and girls (**B**)

The percentile curves for the 9-hole pegboard test are depicted in Figs. 3, 4, and 5 for the right, the left, and both hands, respectively. Boys (Panels A) and girls (Panels B) demonstrated decreasing scores in the time taken to perform the task, indicating a better performance as children age. In both right and left hands, there was a slight tendency for girls to better perform than boys.

The corresponding percentile tables including the age- and sex-specific distribution parameters (*μ*, *σ*, *ν*, *τ*) for the 9-hole pegboard test are available as supplementary files (Supplementary file 2—Tables S3 to S8). The diagnostics plots and the selected modelling parameters for the final models are also available as supplementary files (Supplementary file 1—Figs. 3 to 8).

The results showed that all models provided a good fit to the data, with quantile residuals following a standard normal distribution: means close to 0; variances close to 1; coefficients of skewness close to 0; Filliben correlation coefficients close to 1, as presented in the Supplementary file 1.

## Discussion

This study provides age- and sex-specific percentile curves for STuG and 9-hole pegboard tests for children from different cultural and economic contexts, addressing a significant gap in the extant literature base. The curves and tables provided in this study may enable the assessment of children’s gross and fine motor skills relative to healthy peers and help identify deviations from typical motor development. In conditions like lack of age-appropriate stimulation, or neurodevelopmental deficits, such deviations may provide insights into children´s healthy development.

The STuG test is a version that combines characteristics of two distinct tests: (a) the Timed Up and Go (TUG) test, that is reliable and feasible for application in clinical and non-clinical individuals across the lifespan [23]; and (b) the supine-to-stand (STS) test, conceptualized as a combined assessment of flexibility [24], strength [25], locomotion, and balance [26]. The component sequences of the STS were moderately to strongly correlated with each other (*r* = 0.387 to 0.791) and inversely correlated with the STS time in 3- to 5-year-old children, providing preliminary evidence of its use as a measure across the lifespan [27]. Other authors have also demonstrated that the time it takes to rise from the ground is linked to more advanced movement patterns [27, 28].

The capacity to manipulate one’s center of mass and extremities to rise from the floor, run, and change direction is employed in a variety of functional and recreational activities, including sports and exercise. The use of the STuG test as a product measure of gross motor skills in settings such as early child education centers and preschools aligns with the SUNRISE International Study aims of providing an easy, low-cost, time- and labor-efficient protocol, to fulfill the gap in the literature comprising children´s motor development in LMICs.

In the current study, the centile curves presented for the STuG depicted a decreasing trend as children age, reflecting their greater ability in performing the task with age. Nonetheless, at the oldest age groups (i.e., 54 to 60 months of age), the rank values for girls highlighted a slight stability that might suggest a possible acquisition of motor patterns related to the task performed. Future studies should explore a broader age range to confirm this trend and to identify the age of a subsequent decline in the time to perform the task.

Previous studies have published norms for the 9-hole pegboard test for US children aged 5 to10 years [29]; for Korean children aged 7 to 12 years [30]; and for US children and adolescents aged 4 to19 years [31]. The most recent centile curves were proposed by Wang and colleagues [14], who pooled data from 4319 US individuals to present centile curves for the 9-hole pegboard test across the life span. These curves included data from 331 children aged 3 and 4 years. Similar to the current study, the centile curves proposed by Wang et al. [14] showed a large variation for children in this age range. In fact, the centile curves presented by Wang et al. in 2015 show slightly better time-performance for the task than the ones presented in the current study. It is important to consider that since this study, the global pandemic caused by COVID-19 has drastically increased overall electronic screen-based media use among children, including preschoolers [32]. Although speculative, this epidemiological fact may be seen as a partial justification, as screen use is positively associated with preschoolers’ fine motor skills [33].

The Wang et al. [14] curves suggested a tendency for girls to perform better than boys in the 9-hole pegboard test. Girls and boys are exposed to distinct neuromaturational processes and environmental affordances. Previous research suggests that sex-specific brain development, with certain neural regions predisposing boys to dynamic movement-based activities and girls toward nurturing behaviors [34]. Consequently, fine motor skills may develop earlier in girls than in boys. Empirical evidence supports this hypothesis, as a prior study with 4- to 11-year-old children demonstrated that young girls have greater fine motor skills required in activities demanding a high degree of precision [35]. Moreover, during middle childhood, girls tend to display greater speed and coordination in fine motor skills than boys [36]. Also, some cultural settings from where data provided could lead girls to more household and manual activities, while they might not be so challenged or afforded with opportunities to develop gross skills, when compared to boys. Although previous research suggests that developmental patterns may differ between boys and girls, the present study did not include a formal statistical comparison between the sex-specific curves. Therefore, any discussion of apparent differences should not be interpreted as evidence of inherent or fixed sex-based differences, but rather as a description of observed patterns that may vary across populations and contexts.

Finally, the percentile curves developed by Wang et al. [14], derived from a multiethnic sample of US children, categorized performance according to age in years and results in the dominant and non-dominant hands. While this could be seen as an advantage, especially considering the lifespan spectrum covered by the study, it is less informative for understanding how skilled children are, as fine motor skills change rapidly during early childhood. Hand dominance may still be emerging during early childhood. Although, the functional interpretation of left–right differences should therefore be approached cautiously, the approach used in the current study (i.e., grouping children into 3-month age intervals, presenting hand-specific scores, and including a combined score of both hands) may provide a more comprehensive characterization of fine motor skills performance across the age range. This strategy may also offer useful descriptive information for future investigations examining developmental patterns of manual dexterity. Additionally, the summed pegboard score provides a broader indicator of overall fine motor skills performance and supports interpretation from a more global public health perspective, allowing for a fair representation of developmental patterns across diverse cultural contexts, including children that are not yet in formal educational settings.

Additional points that should be acknowledged are that the percentile curves were derived from pooled multinational data without explicitly modelling the hierarchical structure of the dataset (i.e., children nested within countries, and in some cases within study sites or ECEC centers). Also, the potential sampling biases in certain countries are a clear limitation that may influence the findings. This pooled approach increases the ecological breadth and applicability of the proposed percentile curves. It was adopted to maximize sample size across narrow age bands and to generate harmonized cross-cultural reference values within the scope of the SUNRISE International Study. However, it may also mask country-specific developmental patterns influenced by contextual factors such as educational systems, environmental affordances, urbanization, socioeconomic conditions, and opportunities for movement and play. Therefore, the curves presented should not be interpreted as normative standards for any single population, but rather as harmonized reference values generated within the scope of the SUNRISE International Study. To manage these, we employed an unweighted approach that treated each observation equally, regardless of country of origin. This was adopted due to the absence of population weights and to maintain consistency across countries. The GAMLSS procedure used is particularly suited for constructing percentile curves in the presence of heteroscedasticity and non-normality and does not require homogeneity of variance or symmetry assumptions, accommodating complex growth patterns and distributional shifts, making it a robust choice given the heterogeneity in the data. Country-level weighting was not applied because the participating samples were not obtained under a common probabilistic sampling framework and no predefined target population distribution was available. Applying post hoc weights would therefore require arbitrary assumptions and could give disproportionate influence on smaller, less stable country-specific samples, increasing sampling variability and potentially introducing additional bias.

Consequently, the resulting curves should be interpreted as international SUNRISE-based reference values rather than country-specific normative standards.

The age- and sex-specific percentile curves described cover children from different cultural, social, economic, and environmental contexts, which clearly reflects the variability of children’s motor development. Future studies could use the centile curves and tables provided in this study to enable the assessment of 3–4-year-old children’s gross and fine motor skills relative to healthy peers, helping to identify deviations from typical motor development.

From a public health perspective, these curves can track population-level motor skills trends, highlight at-risk groups of children, and support public health action and intervention planning across SUNRISE countries.

Future studies with larger and more balanced samples across countries should consider multilevel or hierarchical modelling approaches to better account for clustering and to quantify the extent to which country- or site-level factors influence motor performance trajectories. In addition, replication in independent samples, ideally from countries not included in the present analyses and using the same harmonized assessment protocols, would help determine the stability, transportability, and generalizability of the proposed curves across diverse populations and settings.

## Conclusion

This study provides novel, cross-cultural percentile curves for gross and fine motor skills in early childhood, within the scope of the SUNRISE International Study. Derived from data collected across six geographically, culturally, and economically diverse countries, these curves provide an initial harmonized reference for describing children’s gross and fine motor skills performance, supporting practitioners, researchers, and stakeholders in interpreting motor development patterns. It is recommended that the findings be used to advocate for periodic updates to the percentile curves developed using representative datasets, ensuring references for fine and gross motor skills remain relevant and actionable.

## Supplementary Information

Below is the link to the electronic supplementary material.

Supplementary Material File 1 (DOCX 2.20 MB)

Supplementary Material File 2 (DOCX 65.0 KB)

## Data Availability

The data supporting the findings of this study cannot be publicly shared. However, the data will be made available upon reasonable request from the corresponding author.
