# Supplementary material for: Age- and sex-specific percentile curves for gross and fine motor skills in early childhood: an analysis from the SUNRISE International Study
Source: Eur J Pediatr. 2026 Jul 21;185(8):593. doi: 10.1007/s00431-026-07249-y (PMC13388636; doi:10.1007/s00431-026-07249-y)
Supplement: Supplementary file 1 — Supplementary Material File 1 (DOCX 2.20 MB) [file 431_2026_7249_MOESM1_ESM.docx]

**SUPPLEMENTARY FILE 1 – Fit indexes**

| **STUG** |
| --- |

**Boys**


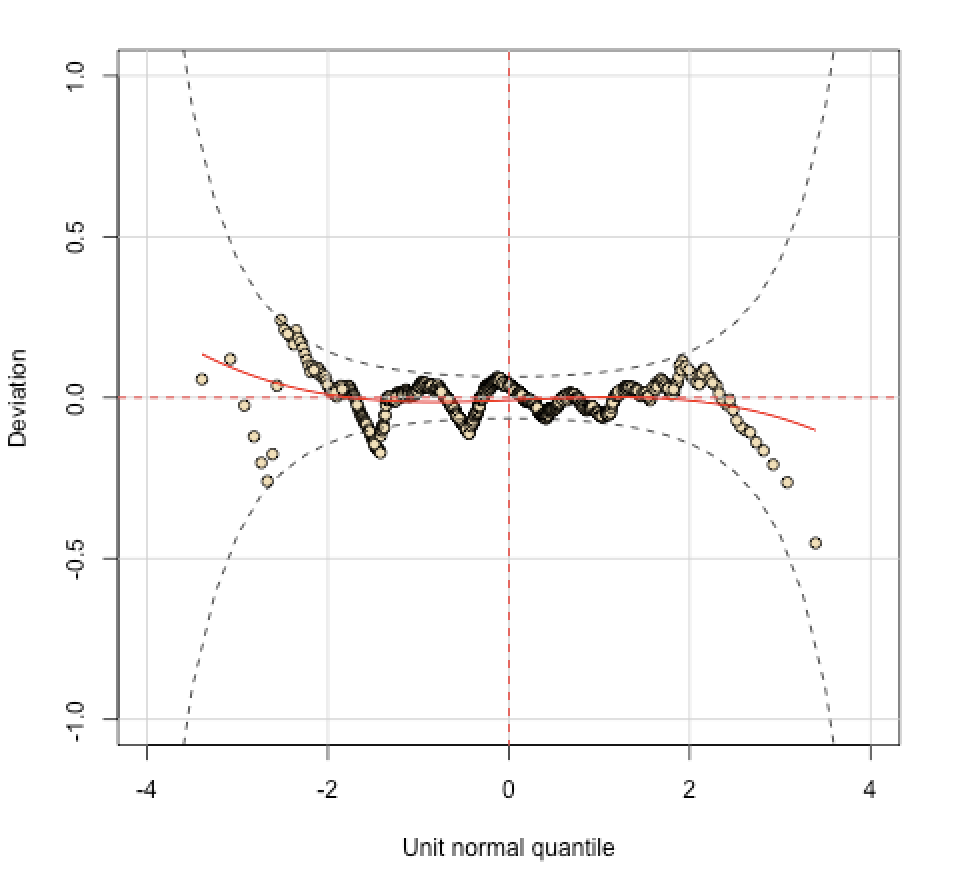


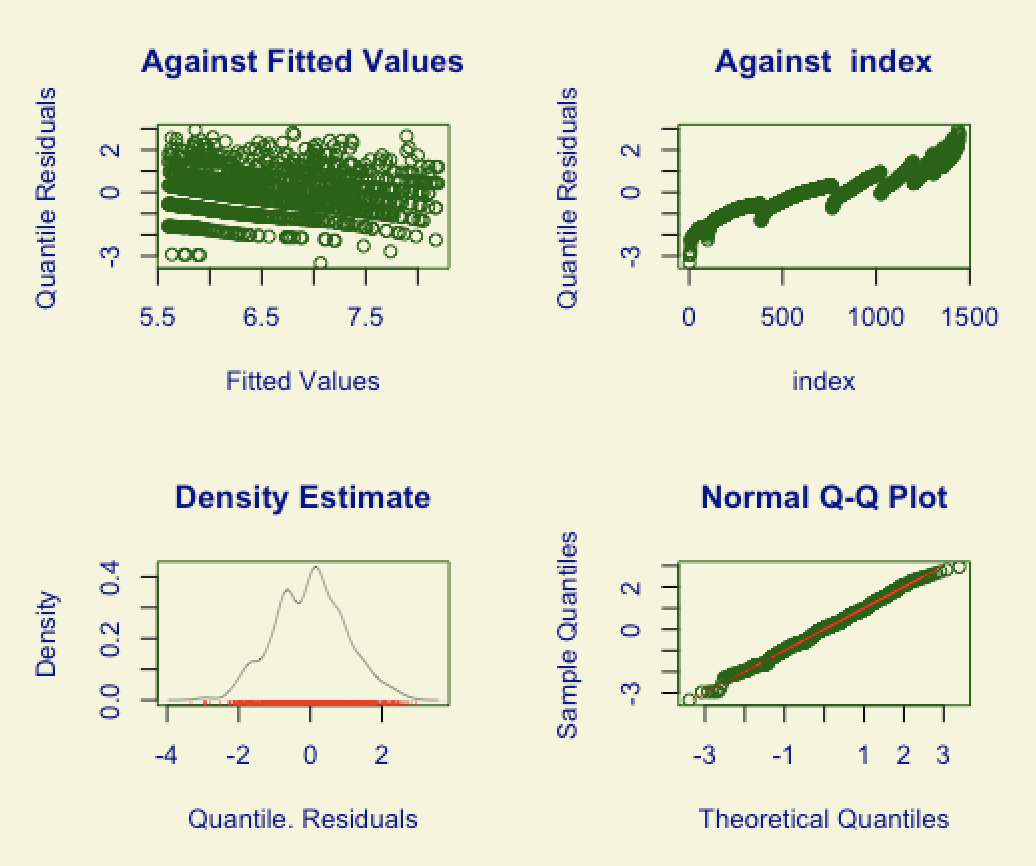


**
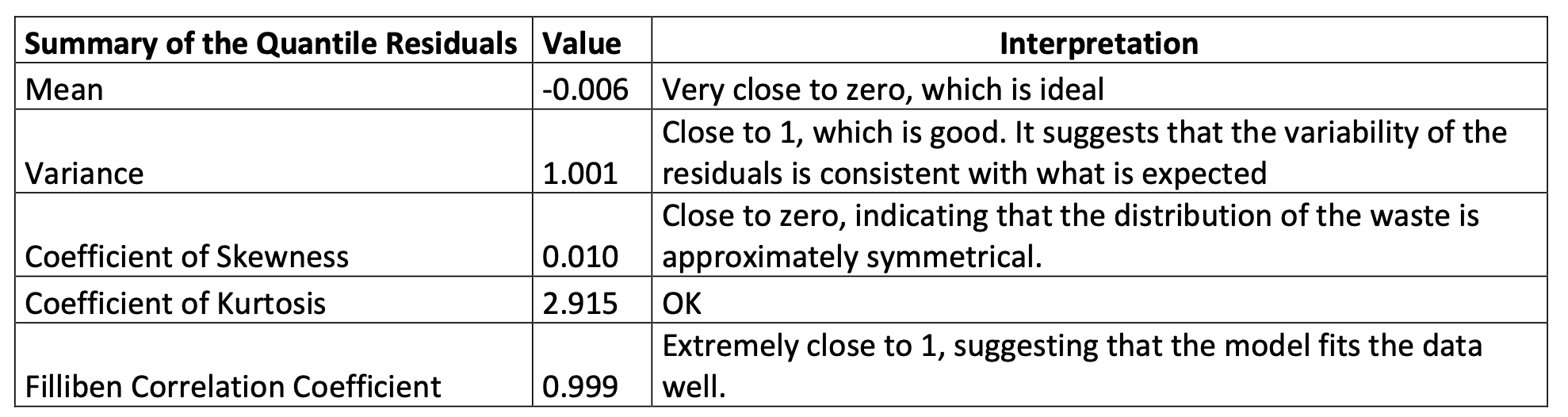
**

**Girls**


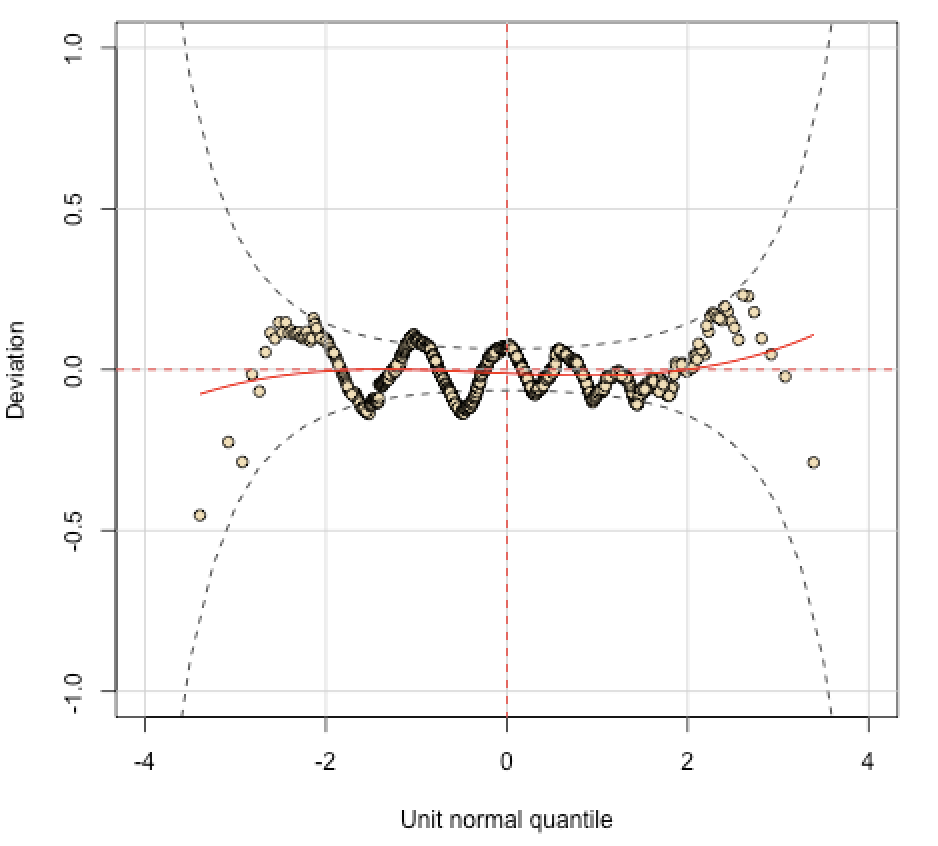


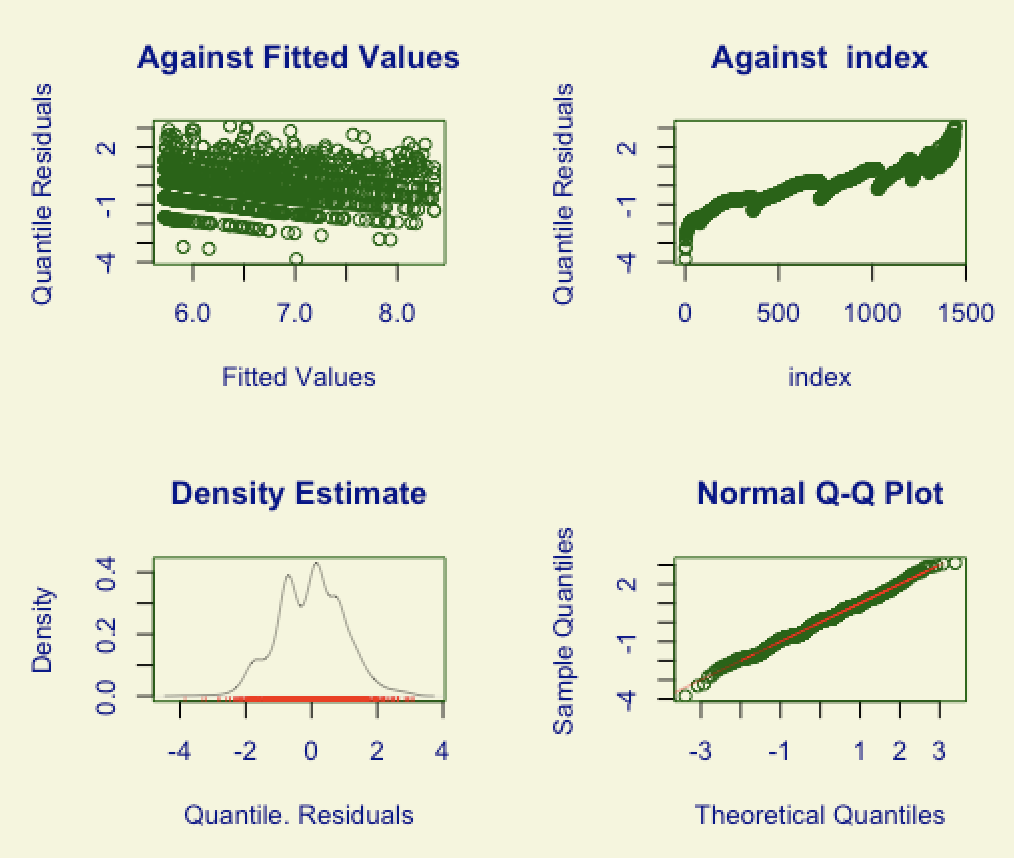


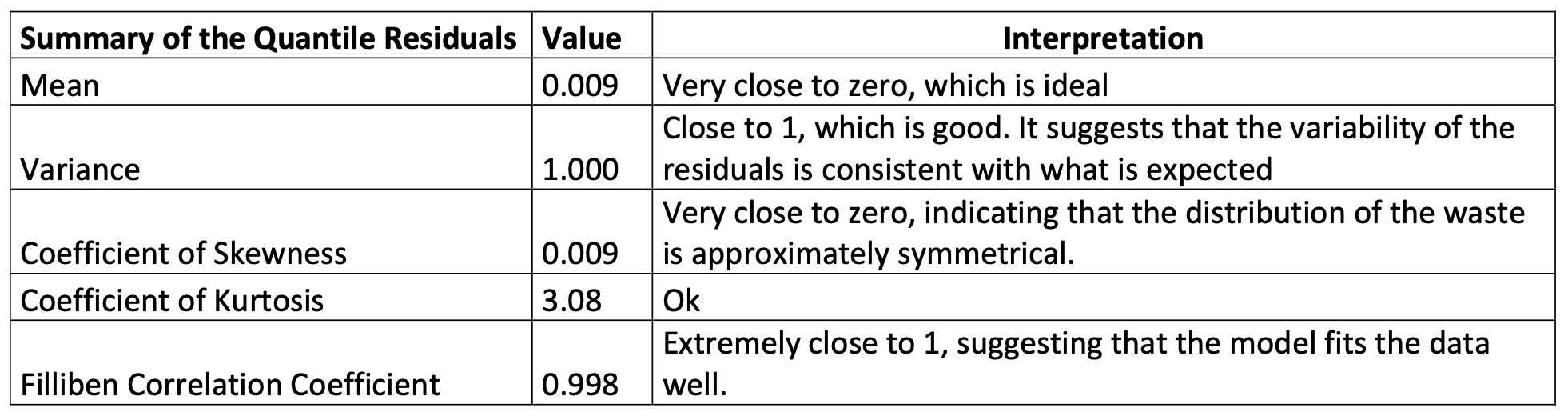


| **PEGBOARD** |
| --- |

**LEFT HAND**

**Boys**


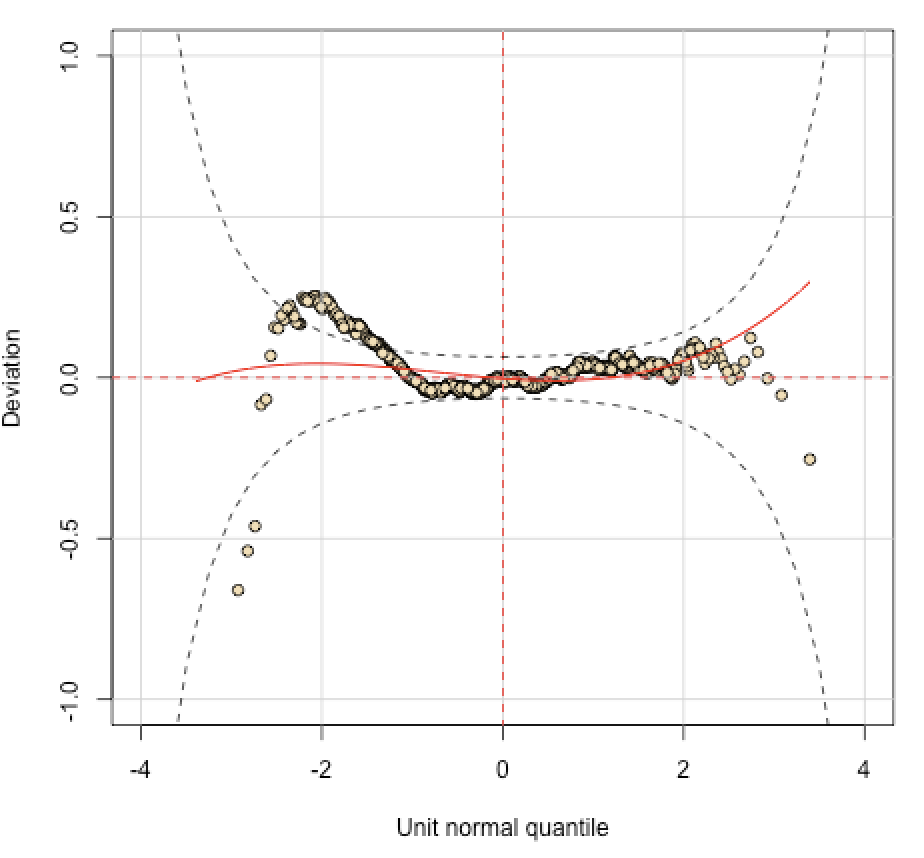


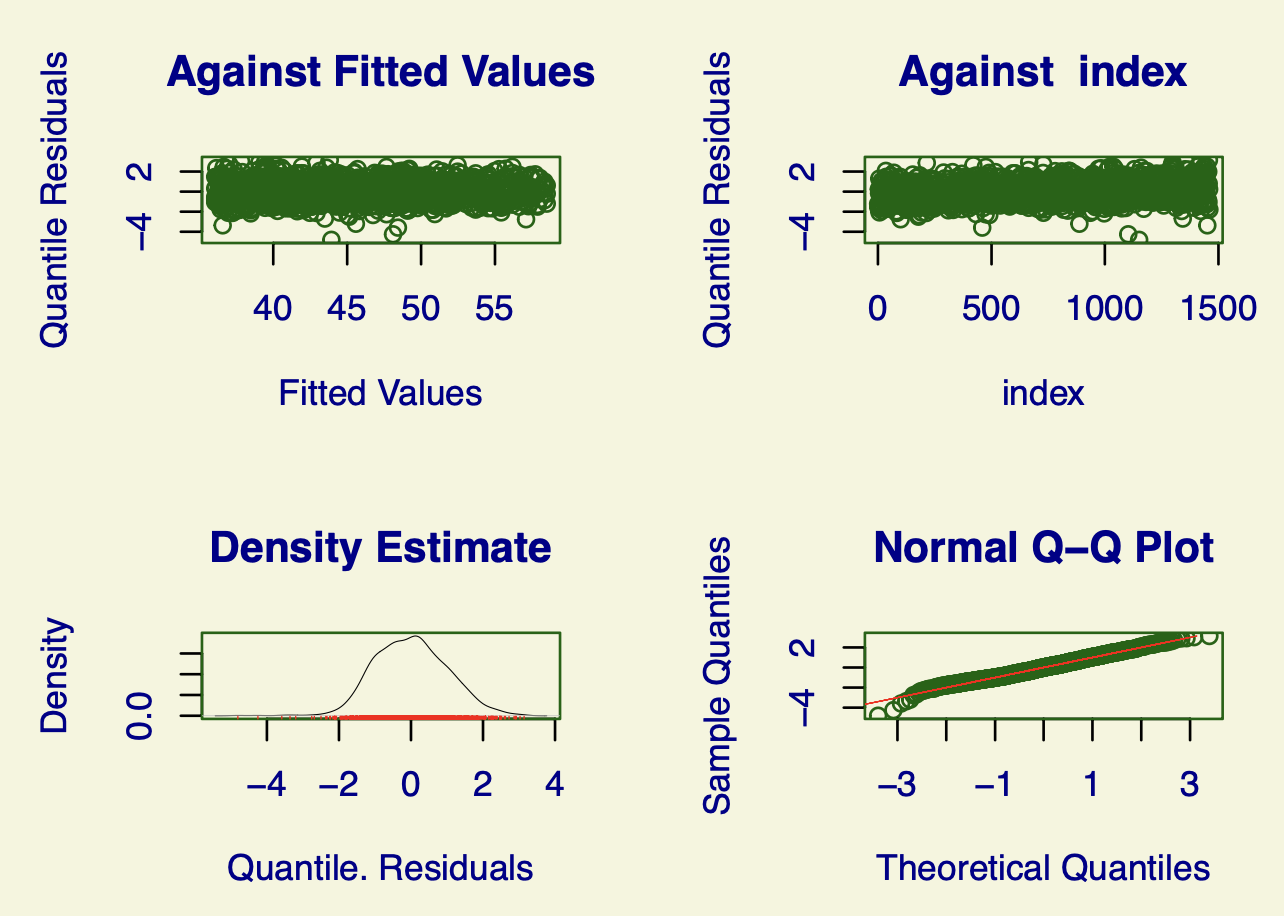


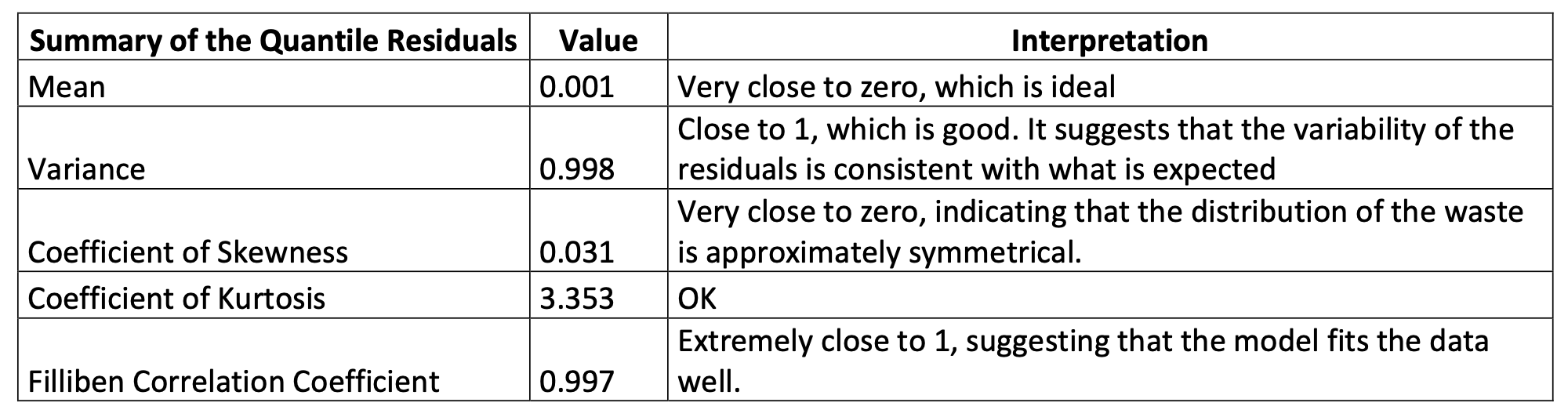


**LEFT HAND**

**Girls**


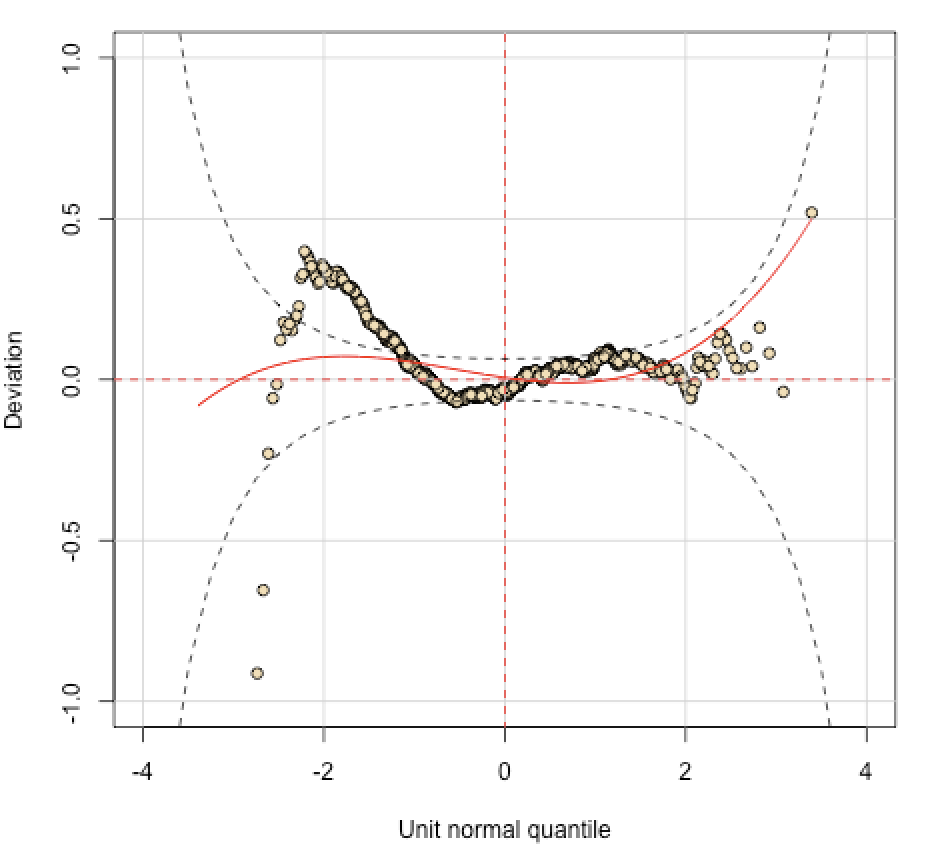


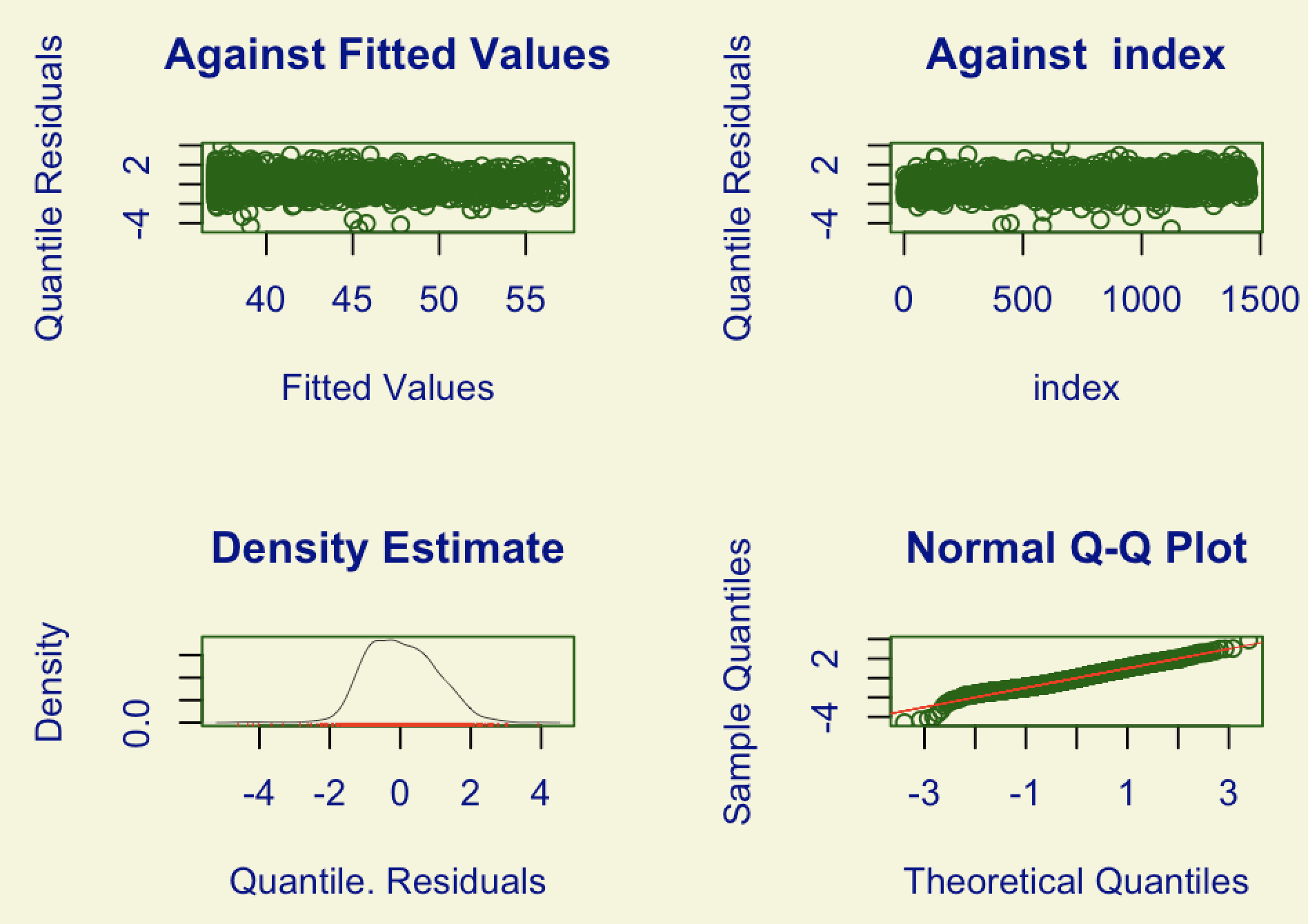


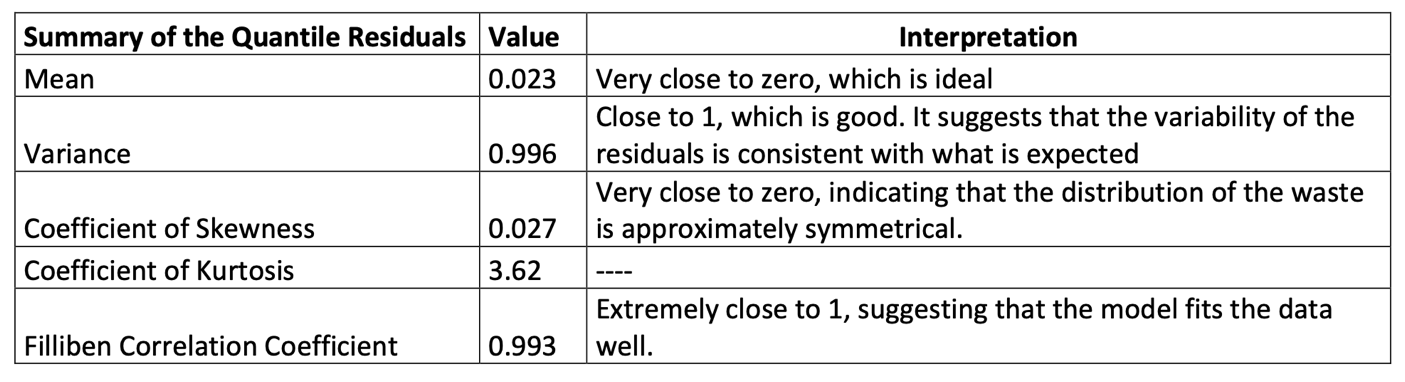


**RIGHT HAND**

**Boys**

**
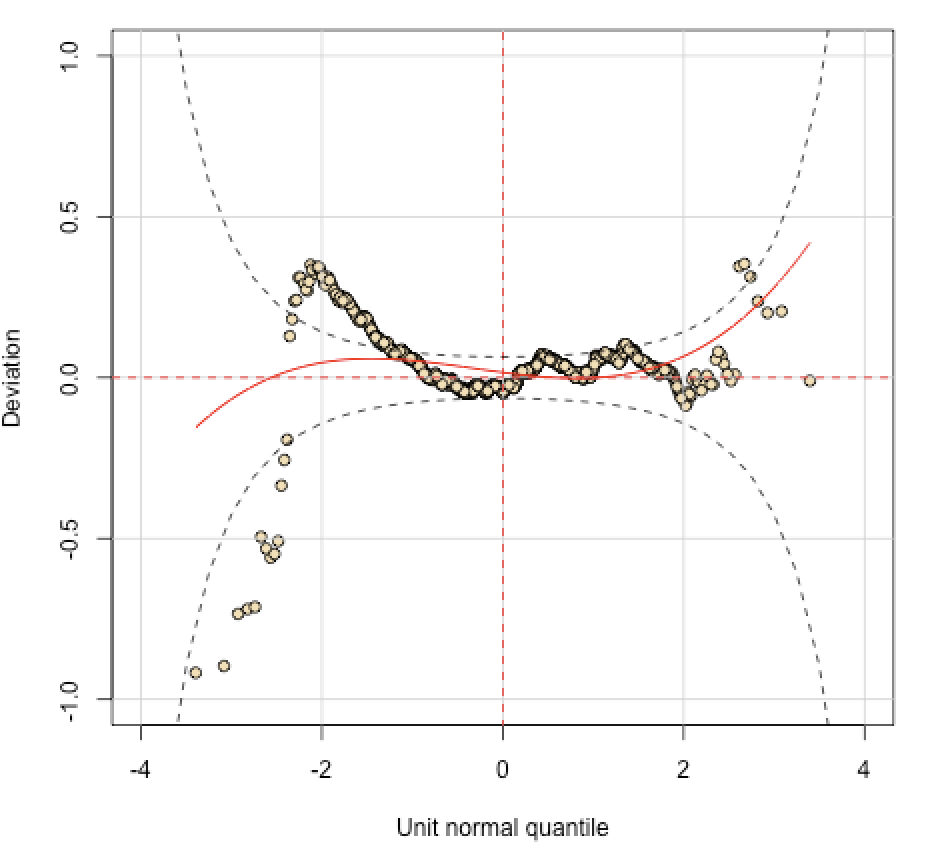
**


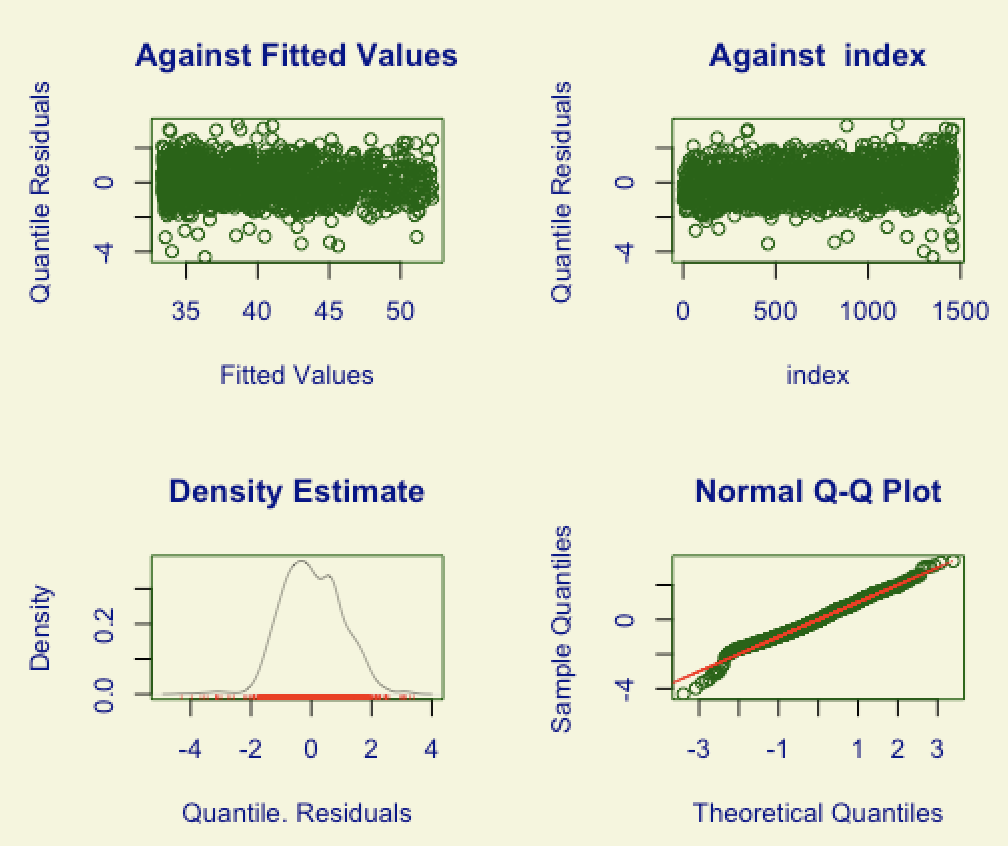


**
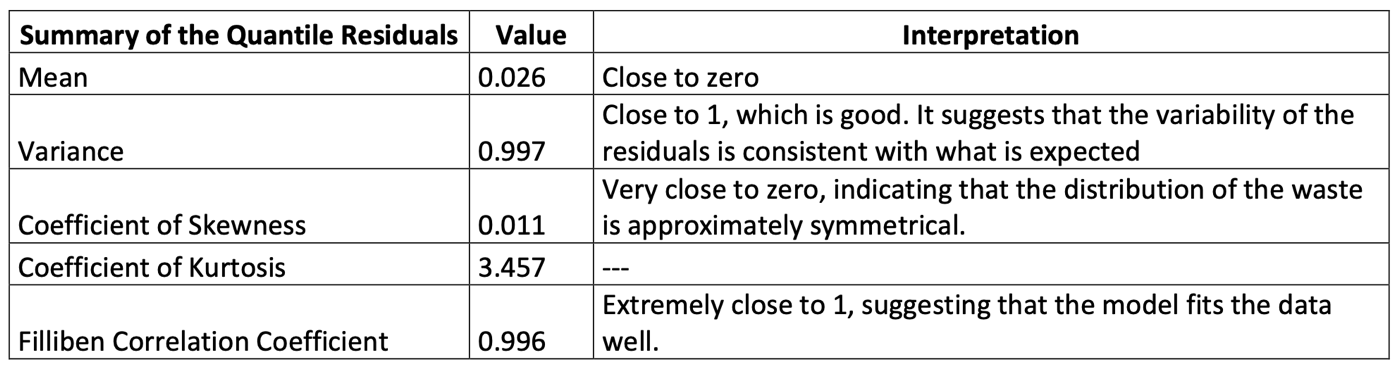
**

**RIGHT HAND**

**Girls**

**
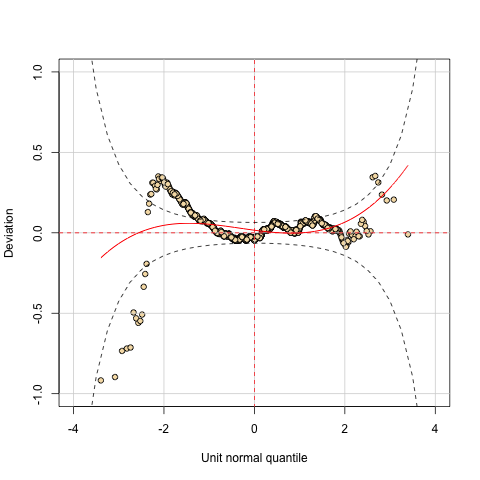
**

**
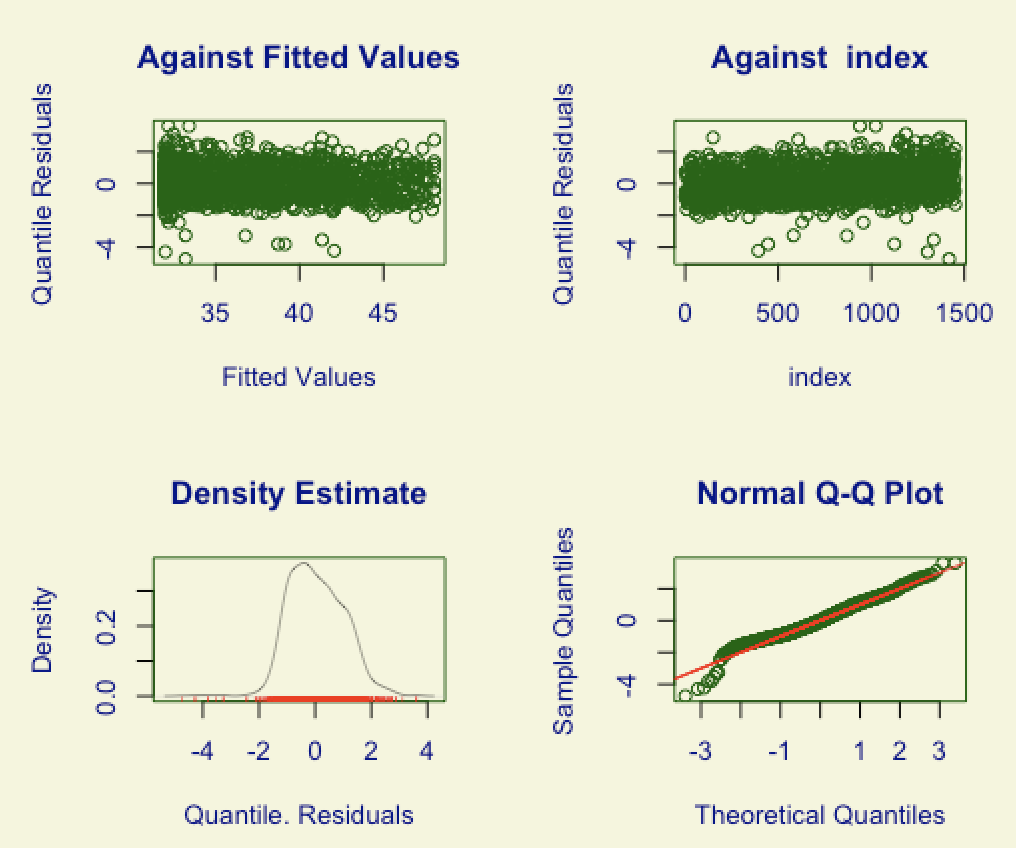
**

**
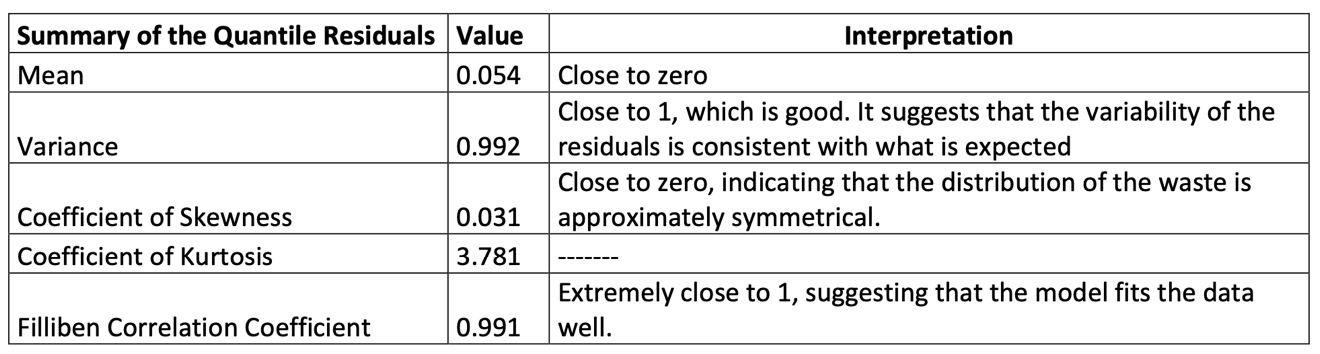
**

**BOTH HANDS**

**Boys**

**
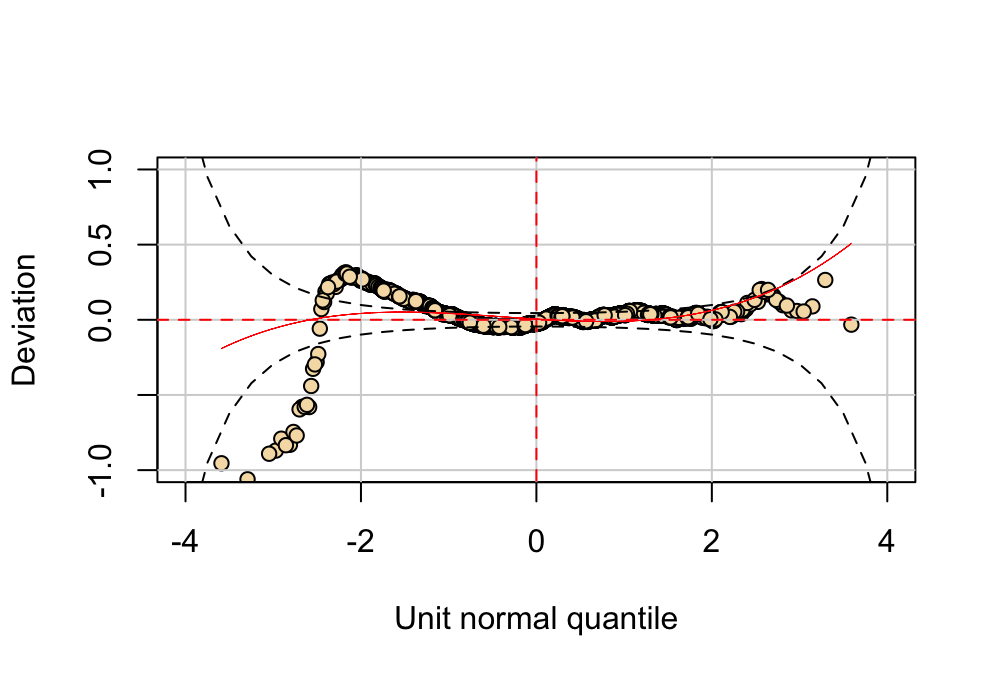
**

**
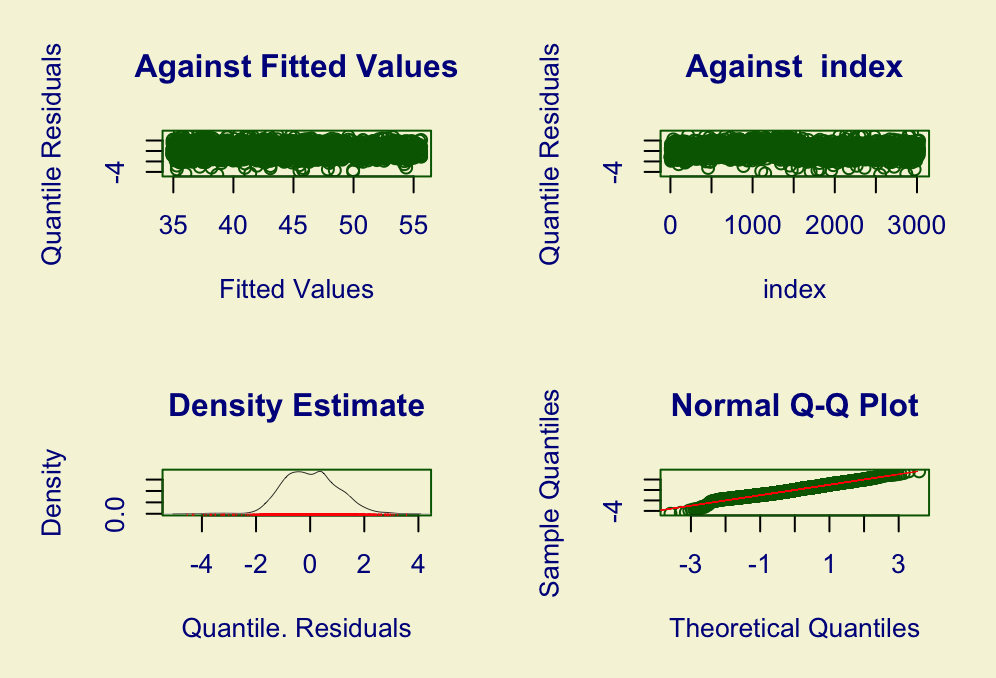
**

| **Summary of the Quantile Residuals** | **Value** | **Interpretation** |
| --- | --- | --- |
| Mean | 0.099 | Very close to zero, which is ideal |
| Variance | 0.998 | Close to 1, which is good. It suggests that the variability of the residuals is consistent with what is expected |
| Coefficient of Skewness | 0.031 | Very close to zero, indicating that the distribution of the waste is approximately symmetrical. |
| Coefficient of Kurtosis | 3.353 | OK |
| Filliben Correlation Coefficient | 0.996 | Extremely close to 1, suggesting that the model fits the data well. |

**BOTH HANDS**

**Girls**

**
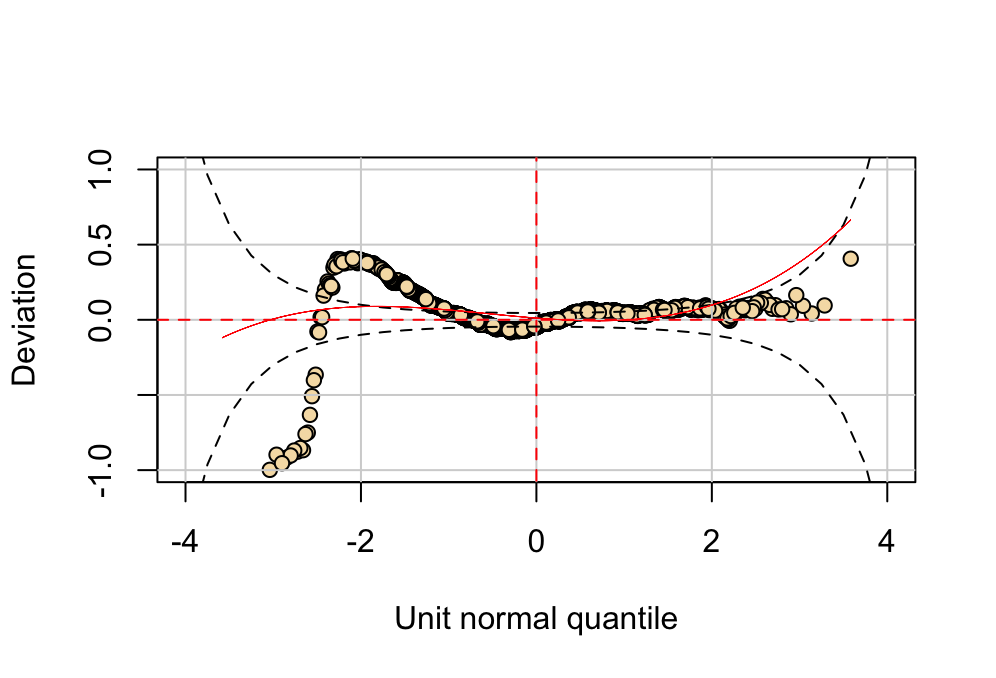
**

**
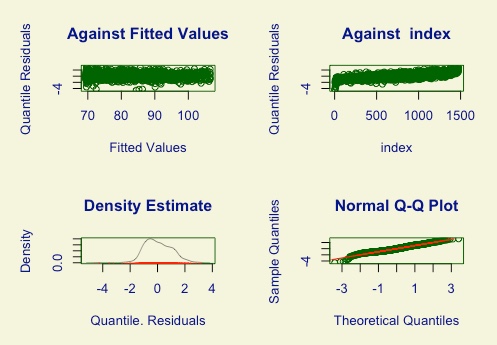
**

| **Summary of the Quantile Residuals** | **Value** | **Interpretation** |
| --- | --- | --- |
| Mean | 0.023 | Very close to zero, which is ideal |
| Variance | 0.996 | Close to 1, which is good. It suggests that the variability of the residuals is consistent with what is expected |
| Coefficient of Skewness | 0.027 | Very close to zero, indicating that the distribution of the waste is approximately symmetrical. |
| Coefficient of Kurtosis | 3.62 | ---- |
| Filliben Correlation Coefficient | 0.993 | Extremely close to 1, suggesting that the model fits the data well. |
