# Supplementary material for: Age- and sex-specific percentile curves for gross and fine motor skills in early childhood: an analysis from the SUNRISE International Study
Source: Eur J Pediatr. 2026 Jul 21;185(8):593. doi: 10.1007/s00431-026-07249-y (PMC13388636; doi:10.1007/s00431-026-07249-y)
Supplement: Supplementary file 2 — Supplementary Material File 2 (DOCX 65.0 KB) [file 431_2026_7249_MOESM2_ESM.docx]

**SUPPLEMENTARY FILE 2 – Percentile Values**

| **STuG** |
| --- |

The percentile tables including the age- and sex-specific distribution parameters (μ,σ,ν,τ) for the STuG are available as a supplement (Tables S1, and S2).

**S1. Percentile values for STuG in boys**

| AGE_M | mu | sigma | nu | tau | P10 | P25 | P50 | P75 | P90 |
| --- | --- | --- | --- | --- | --- | --- | --- | --- | --- |
| 36 | 8.2 | 0.2 | -0.5 | 1.6 | 6.2 | 7.1 | 8.2 | 9.6 | 11.3 |
| 37 | 8.0 | 0.2 | -0.5 | 1.6 | 6.0 | 6.9 | 8.0 | 9.4 | 11.2 |
| 38 | 7.8 | 0.3 | -0.5 | 1.6 | 5.8 | 6.7 | 7.8 | 9.2 | 11.1 |
| 39 | 7.6 | 0.3 | -0.5 | 1.6 | 5.6 | 6.5 | 7.6 | 9.1 | 11.0 |
| 40 | 7.5 | 0.3 | -0.5 | 1.6 | 5.5 | 6.4 | 7.5 | 9.0 | 10.9 |
| 41 | 7.4 | 0.3 | -0.5 | 1.6 | 5.3 | 6.2 | 7.4 | 8.8 | 10.8 |
| 42 | 7.3 | 0.3 | -0.5 | 1.6 | 5.2 | 6.1 | 7.3 | 8.7 | 10.7 |
| 43 | 7.2 | 0.3 | -0.5 | 1.6 | 5.1 | 6.0 | 7.2 | 8.6 | 10.6 |
| 44 | 7.0 | 0.3 | -0.5 | 1.6 | 5.1 | 5.9 | 7.0 | 8.5 | 10.5 |
| 45 | 6.9 | 0.3 | -0.5 | 1.6 | 5.0 | 5.9 | 6.9 | 8.3 | 10.2 |
| 46 | 6.8 | 0.3 | -0.5 | 1.6 | 5.0 | 5.8 | 6.8 | 8.2 | 9.9 |
| 47 | 6.7 | 0.3 | -0.5 | 1.6 | 4.9 | 5.7 | 6.7 | 8.0 | 9.6 |
| 48 | 6.5 | 0.3 | -0.5 | 1.6 | 4.8 | 5.6 | 6.5 | 7.7 | 9.3 |
| 49 | 6.4 | 0.3 | -0.5 | 1.6 | 4.8 | 5.5 | 6.4 | 7.6 | 9.0 |
| 50 | 6.3 | 0.3 | -0.5 | 1.6 | 4.7 | 5.4 | 6.3 | 7.4 | 8.8 |
| 51 | 6.2 | 0.2 | -0.5 | 1.6 | 4.6 | 5.3 | 6.2 | 7.2 | 8.6 |
| 52 | 6.1 | 0.2 | -0.5 | 1.6 | 4.5 | 5.2 | 6.1 | 7.1 | 8.5 |
| 53 | 6.0 | 0.2 | -0.5 | 1.6 | 4.5 | 5.2 | 6.0 | 7.0 | 8.4 |
| 54 | 5.9 | 0.2 | -0.5 | 1.6 | 4.4 | 5.1 | 5.9 | 7.0 | 8.3 |
| 55 | 5.9 | 0.2 | -0.5 | 1.6 | 4.4 | 5.0 | 5.9 | 6.9 | 8.2 |
| 56 | 5.8 | 0.2 | -0.5 | 1.6 | 4.4 | 5.0 | 5.8 | 6.8 | 8.0 |
| 57 | 5.7 | 0.2 | -0.5 | 1.6 | 4.3 | 5.0 | 5.7 | 6.7 | 7.9 |
| 58 | 5.7 | 0.2 | -0.5 | 1.6 | 4.3 | 4.9 | 5.7 | 6.6 | 7.8 |
| 59 | 5.6 | 0.2 | -0.5 | 1.6 | 4.3 | 4.9 | 5.6 | 6.6 | 7.7 |
| 60 | 5.6 | 0.2 | -0.5 | 1.6 | 4.3 | 4.9 | 5.6 | 6.5 | 7.6 |

**S2. Percentile values for STuG in girls**

| AGE_M | mu | sigma | nu | tau | P10 | P25 | P50 | P75 | P90 |
| --- | --- | --- | --- | --- | --- | --- | --- | --- | --- |
| 36 | 8.4 | 0.3 | -0.6 | 1.6 | 6.0 | 7.1 | 8.4 | 10.1 | 12.5 |
| 37 | 8.2 | 0.3 | -0.6 | 1.6 | 5.9 | 6.9 | 8.2 | 9.8 | 12.1 |
| 38 | 8.0 | 0.3 | -0.6 | 1.6 | 5.8 | 6.8 | 8.0 | 9.6 | 11.7 |
| 39 | 7.8 | 0.3 | -0.6 | 1.6 | 5.7 | 6.6 | 7.8 | 9.3 | 11.3 |
| 40 | 7.6 | 0.3 | -0.6 | 1.6 | 5.6 | 6.5 | 7.6 | 9.1 | 11.0 |
| 41 | 7.4 | 0.3 | -0.6 | 1.6 | 5.5 | 6.4 | 7.4 | 8.8 | 10.6 |
| 42 | 7.3 | 0.3 | -0.6 | 1.6 | 5.4 | 6.2 | 7.3 | 8.6 | 10.3 |
| 43 | 7.1 | 0.3 | -0.6 | 1.6 | 5.3 | 6.1 | 7.1 | 8.4 | 10.1 |
| 44 | 7.0 | 0.2 | -0.6 | 1.6 | 5.3 | 6.0 | 7.0 | 8.2 | 9.8 |
| 45 | 6.9 | 0.2 | -0.6 | 1.6 | 5.2 | 5.9 | 6.9 | 8.1 | 9.6 |
| 46 | 6.8 | 0.2 | -0.6 | 1.6 | 5.1 | 5.9 | 6.8 | 8.0 | 9.5 |
| 47 | 6.7 | 0.2 | -0.6 | 1.6 | 5.0 | 5.8 | 6.7 | 7.8 | 9.3 |
| 48 | 6.6 | 0.2 | -0.6 | 1.6 | 5.0 | 5.7 | 6.6 | 7.7 | 9.2 |
| 49 | 6.5 | 0.2 | -0.6 | 1.6 | 4.9 | 5.6 | 6.5 | 7.6 | 9.1 |
| 50 | 6.4 | 0.2 | -0.6 | 1.6 | 4.8 | 5.5 | 6.4 | 7.5 | 8.9 |
| 51 | 6.2 | 0.2 | -0.6 | 1.6 | 4.7 | 5.4 | 6.2 | 7.3 | 8.7 |
| 52 | 6.1 | 0.2 | -0.6 | 1.6 | 4.6 | 5.3 | 6.1 | 7.2 | 8.5 |
| 53 | 6.0 | 0.2 | -0.6 | 1.6 | 4.6 | 5.2 | 6.0 | 7.0 | 8.3 |
| 54 | 5.9 | 0.2 | -0.6 | 1.6 | 4.5 | 5.1 | 5.9 | 6.9 | 8.2 |
| 55 | 5.9 | 0.2 | -0.6 | 1.6 | 4.5 | 5.1 | 5.9 | 6.8 | 8.0 |
| 56 | 5.8 | 0.2 | -0.6 | 1.6 | 4.5 | 5.1 | 5.8 | 6.8 | 7.9 |
| 57 | 5.8 | 0.2 | -0.6 | 1.6 | 4.4 | 5.0 | 5.8 | 6.7 | 7.9 |
| 58 | 5.8 | 0.2 | -0.6 | 1.6 | 4.4 | 5.0 | 5.8 | 6.7 | 7.9 |
| 59 | 5.7 | 0.2 | -0.6 | 1.6 | 4.4 | 5.0 | 5.7 | 6.7 | 7.9 |
| 60 | 5.7 | 0.2 | -0.6 | 1.6 | 4.3 | 5.0 | 5.7 | 6.7 | 7.9 |

| **PEGBOARD** |
| --- |

The percentile tables including the age- and sex-specific distribution parameters (μ,σ,ν,τ) for the Pegboard test are available as a supplement (Tables S3, S4, S5, S6, S7, and S8).

**S3. Percentile values for Pegboard – left hand in boys**

| AGE_M | mu | sigma | nu | tau | P3 | P10 | P25 | P50 | P75 | P90 | P97 |
| --- | --- | --- | --- | --- | --- | --- | --- | --- | --- | --- | --- |
| 36 | 58.5 | 0.2 | 0.1 | 1.5 | 36.0 | 43.0 | 50.4 | 58.5 | 67.8 | 78.9 | 93.1 |
| 37 | 57.2 | 0.2 | 0.1 | 1.5 | 35.1 | 42.0 | 49.2 | 57.2 | 66.3 | 77.2 | 91.2 |
| 38 | 55.9 | 0.2 | 0.1 | 1.5 | 34.3 | 41.0 | 48.1 | 55.9 | 64.9 | 75.5 | 89.2 |
| 39 | 54.7 | 0.2 | 0.1 | 1.5 | 33.6 | 40.2 | 47.1 | 54.7 | 63.4 | 73.8 | 87.1 |
| 40 | 53.5 | 0.2 | 0.1 | 1.5 | 33.0 | 39.4 | 46.1 | 53.5 | 62.0 | 72.1 | 85.1 |
| 41 | 52.4 | 0.2 | 0.1 | 1.5 | 32.3 | 38.6 | 45.1 | 52.4 | 60.7 | 70.5 | 83.1 |
| 42 | 51.3 | 0.2 | 0.1 | 1.5 | 31.6 | 37.8 | 44.2 | 51.3 | 59.4 | 69.1 | 81.4 |
| 43 | 50.3 | 0.2 | 0.1 | 1.5 | 31.0 | 37.0 | 43.3 | 50.3 | 58.3 | 67.8 | 80.0 |
| 44 | 49.4 | 0.2 | 0.1 | 1.5 | 30.3 | 36.2 | 42.5 | 49.4 | 57.2 | 66.6 | 78.6 |
| 45 | 48.4 | 0.2 | 0.1 | 1.5 | 29.7 | 35.6 | 41.7 | 48.4 | 56.2 | 65.4 | 77.2 |
| 46 | 47.5 | 0.2 | 0.1 | 1.5 | 29.3 | 35.0 | 40.9 | 47.5 | 55.0 | 64.0 | 75.5 |
| 47 | 46.6 | 0.2 | 0.1 | 1.5 | 28.8 | 34.4 | 40.2 | 46.6 | 53.8 | 62.5 | 73.6 |
| 48 | 45.6 | 0.2 | 0.1 | 1.5 | 28.3 | 33.7 | 39.4 | 45.6 | 52.7 | 61.1 | 71.9 |
| 49 | 44.7 | 0.2 | 0.1 | 1.5 | 27.8 | 33.1 | 38.6 | 44.7 | 51.6 | 59.8 | 70.2 |
| 50 | 43.7 | 0.2 | 0.1 | 1.5 | 27.4 | 32.5 | 37.9 | 43.7 | 50.5 | 58.4 | 68.6 |
| 51 | 42.8 | 0.2 | 0.1 | 1.5 | 27.0 | 31.9 | 37.1 | 42.8 | 49.3 | 56.9 | 66.7 |
| 52 | 41.9 | 0.2 | 0.1 | 1.5 | 26.6 | 31.5 | 36.4 | 41.9 | 48.1 | 55.4 | 64.7 |
| 53 | 41.1 | 0.2 | 0.1 | 1.5 | 26.4 | 31.1 | 35.9 | 41.1 | 46.9 | 53.9 | 62.6 |
| 54 | 40.3 | 0.2 | 0.1 | 1.5 | 26.3 | 30.7 | 35.3 | 40.3 | 45.9 | 52.4 | 60.7 |
| 55 | 39.5 | 0.2 | 0.1 | 1.5 | 26.1 | 30.4 | 34.8 | 39.5 | 44.8 | 51.0 | 58.8 |
| 56 | 38.7 | 0.2 | 0.1 | 1.5 | 25.9 | 30.0 | 34.2 | 38.7 | 43.7 | 49.6 | 56.9 |
| 57 | 37.9 | 0.2 | 0.1 | 1.5 | 25.7 | 29.6 | 33.6 | 37.9 | 42.7 | 48.2 | 55.1 |
| 58 | 37.2 | 0.2 | 0.1 | 1.5 | 25.5 | 29.3 | 33.1 | 37.2 | 41.8 | 47.1 | 53.7 |
| 59 | 36.6 | 0.2 | 0.1 | 1.5 | 25.3 | 28.9 | 32.6 | 36.6 | 41.0 | 46.1 | 52.4 |
| 60 | 36.0 | 0.2 | 0.1 | 1.5 | 25.1 | 28.6 | 32.2 | 36.0 | 40.2 | 45.1 | 51.1 |

**S4. Percentile values for Pegboard – left hand in girls**

| AGE_M | mu | sigma | nu | tau | P3 | P10 | P25 | P50 | P75 | P90 | P97 |
| --- | --- | --- | --- | --- | --- | --- | --- | --- | --- | --- | --- |
| 36 | 57.0 | 0.3 | 0.1 | 1.5 | 34.1 | 41.3 | 48.8 | 57.0 | 66.4 | 77.8 | 92.5 |
| 37 | 55.6 | 0.3 | 0.1 | 1.5 | 33.6 | 40.5 | 47.7 | 55.6 | 64.5 | 75.4 | 89.3 |
| 38 | 54.1 | 0.2 | 0.1 | 1.5 | 33.1 | 39.7 | 46.6 | 54.1 | 62.7 | 73.0 | 86.2 |
| 39 | 52.8 | 0.2 | 0.1 | 1.5 | 32.6 | 38.9 | 45.6 | 52.8 | 61.0 | 70.8 | 83.4 |
| 40 | 51.5 | 0.2 | 0.1 | 1.5 | 31.9 | 38.1 | 44.5 | 51.5 | 59.4 | 68.9 | 81.0 |
| 41 | 50.2 | 0.2 | 0.1 | 1.5 | 31.2 | 37.2 | 43.5 | 50.2 | 57.9 | 67.1 | 78.9 |
| 42 | 49.1 | 0.2 | 0.1 | 1.5 | 30.4 | 36.3 | 42.4 | 49.1 | 56.6 | 65.7 | 77.2 |
| 43 | 48.0 | 0.2 | 0.1 | 1.5 | 29.6 | 35.4 | 41.4 | 48.0 | 55.4 | 64.4 | 75.8 |
| 44 | 47.0 | 0.2 | 0.1 | 1.5 | 28.8 | 34.5 | 40.5 | 47.0 | 54.4 | 63.2 | 74.6 |
| 45 | 46.0 | 0.2 | 0.1 | 1.5 | 28.1 | 33.7 | 39.6 | 46.0 | 53.3 | 62.1 | 73.4 |
| 46 | 45.1 | 0.2 | 0.1 | 1.5 | 27.5 | 33.0 | 38.8 | 45.1 | 52.3 | 60.9 | 72.0 |
| 47 | 44.2 | 0.2 | 0.1 | 1.5 | 27.1 | 32.5 | 38.1 | 44.2 | 51.1 | 59.5 | 70.2 |
| 48 | 43.2 | 0.2 | 0.1 | 1.5 | 26.7 | 31.9 | 37.3 | 43.2 | 49.9 | 57.9 | 68.2 |
| 49 | 42.3 | 0.2 | 0.1 | 1.5 | 26.4 | 31.4 | 36.6 | 42.3 | 48.8 | 56.4 | 66.3 |
| 50 | 41.4 | 0.2 | 0.1 | 1.5 | 26.0 | 30.9 | 36.0 | 41.4 | 47.7 | 55.1 | 64.5 |
| 51 | 40.6 | 0.2 | 0.1 | 1.5 | 25.6 | 30.4 | 35.3 | 40.6 | 46.6 | 53.8 | 63.0 |
| 52 | 39.8 | 0.2 | 0.1 | 1.5 | 25.2 | 29.8 | 34.6 | 39.8 | 45.7 | 52.7 | 61.7 |
| 53 | 39.1 | 0.2 | 0.1 | 1.5 | 24.7 | 29.3 | 34.0 | 39.1 | 44.9 | 51.7 | 60.4 |
| 54 | 38.5 | 0.2 | 0.1 | 1.5 | 24.4 | 28.9 | 33.5 | 38.5 | 44.1 | 50.7 | 59.2 |
| 55 | 37.9 | 0.2 | 0.1 | 1.5 | 24.3 | 28.6 | 33.1 | 37.9 | 43.4 | 49.8 | 58.0 |
| 56 | 37.5 | 0.2 | 0.1 | 1.5 | 24.2 | 28.4 | 32.8 | 37.5 | 42.8 | 49.1 | 57.0 |
| 57 | 37.3 | 0.2 | 0.1 | 1.5 | 24.2 | 28.4 | 32.7 | 37.3 | 42.4 | 48.6 | 56.3 |
| 58 | 37.1 | 0.2 | 0.1 | 1.5 | 24.2 | 28.3 | 32.6 | 37.1 | 42.2 | 48.3 | 55.9 |
| 59 | 37.1 | 0.2 | 0.1 | 1.5 | 24.2 | 28.4 | 32.6 | 37.1 | 42.2 | 48.1 | 55.7 |
| 60 | 37.1 | 0.2 | 0.1 | 1.5 | 24.3 | 28.4 | 32.6 | 37.1 | 42.1 | 48.1 | 55.6 |

**S5. Percentile values for Pegboard – right hand in boys**

| AGE_M | mu | sigma | nu | tau | P3 | P10 | P25 | P50 | P75 | P90 | P97 |
| --- | --- | --- | --- | --- | --- | --- | --- | --- | --- | --- | --- |
| 36 | 52.2 | 0.2 | 0.2 | 1.4 | 32.6 | 39.0 | 45.5 | 52.2 | 59.7 | 68.8 | 80.4 |
| 37 | 51.1 | 0.2 | 0.2 | 1.4 | 32.1 | 38.4 | 44.6 | 51.1 | 58.3 | 67.0 | 78.2 |
| 38 | 50.0 | 0.2 | 0.2 | 1.4 | 31.6 | 37.7 | 43.7 | 50.0 | 56.9 | 65.3 | 76.0 |
| 39 | 48.8 | 0.2 | 0.2 | 1.4 | 31.1 | 37.0 | 42.8 | 48.8 | 55.5 | 63.7 | 74.0 |
| 40 | 47.7 | 0.2 | 0.2 | 1.4 | 30.4 | 36.1 | 41.8 | 47.7 | 54.3 | 62.2 | 72.4 |
| 41 | 46.7 | 0.2 | 0.2 | 1.4 | 29.5 | 35.2 | 40.8 | 46.7 | 53.1 | 61.0 | 71.0 |
| 42 | 45.6 | 0.2 | 0.2 | 1.4 | 28.6 | 34.2 | 39.8 | 45.6 | 52.1 | 60.0 | 70.1 |
| 43 | 44.7 | 0.2 | 0.2 | 1.4 | 27.7 | 33.3 | 38.9 | 44.7 | 51.2 | 59.1 | 69.3 |
| 44 | 43.9 | 0.2 | 0.2 | 1.4 | 26.9 | 32.4 | 38.0 | 43.9 | 50.4 | 58.3 | 68.6 |
| 45 | 43.1 | 0.2 | 0.2 | 1.4 | 26.2 | 31.7 | 37.3 | 43.1 | 49.5 | 57.5 | 67.7 |
| 46 | 42.3 | 0.2 | 0.2 | 1.4 | 25.7 | 31.1 | 36.6 | 42.3 | 48.6 | 56.5 | 66.6 |
| 47 | 41.4 | 0.2 | 0.2 | 1.4 | 25.2 | 30.5 | 35.8 | 41.4 | 47.6 | 55.3 | 65.2 |
| 48 | 40.5 | 0.2 | 0.2 | 1.4 | 24.7 | 29.9 | 35.1 | 40.5 | 46.6 | 54.0 | 63.6 |
| 49 | 39.6 | 0.2 | 0.2 | 1.4 | 24.3 | 29.3 | 34.3 | 39.6 | 45.5 | 52.7 | 61.9 |
| 50 | 38.7 | 0.2 | 0.2 | 1.4 | 23.9 | 28.8 | 33.6 | 38.7 | 44.4 | 51.4 | 60.3 |
| 51 | 37.9 | 0.2 | 0.2 | 1.4 | 23.5 | 28.2 | 33.0 | 37.9 | 43.4 | 50.1 | 58.7 |
| 52 | 37.1 | 0.2 | 0.2 | 1.4 | 23.2 | 27.7 | 32.3 | 37.1 | 42.4 | 48.8 | 57.1 |
| 53 | 36.3 | 0.2 | 0.2 | 1.4 | 22.8 | 27.3 | 31.7 | 36.3 | 41.5 | 47.7 | 55.7 |
| 54 | 35.7 | 0.2 | 0.2 | 1.4 | 22.5 | 26.8 | 31.2 | 35.7 | 40.7 | 46.8 | 54.6 |
| 55 | 35.1 | 0.2 | 0.2 | 1.4 | 22.1 | 26.4 | 30.7 | 35.1 | 40.0 | 45.9 | 53.6 |
| 56 | 34.6 | 0.2 | 0.2 | 1.4 | 21.8 | 26.0 | 30.2 | 34.6 | 39.4 | 45.3 | 52.8 |
| 57 | 34.2 | 0.2 | 0.2 | 1.4 | 21.5 | 25.7 | 29.9 | 34.2 | 39.0 | 44.8 | 52.3 |
| 58 | 33.8 | 0.2 | 0.2 | 1.4 | 21.3 | 25.4 | 29.6 | 33.8 | 38.6 | 44.4 | 51.9 |
| 59 | 33.6 | 0.2 | 0.2 | 1.4 | 21.1 | 25.2 | 29.3 | 33.6 | 38.3 | 44.0 | 51.4 |
| 60 | 33.3 | 0.2 | 0.2 | 1.4 | 21.0 | 25.0 | 29.1 | 33.3 | 38.0 | 43.7 | 51.0 |

**S6. Percentile values for Pegboard – right hand in girls**

| AGE_M | mu | sigma | nu | tau | P3 | P10 | P25 | P50 | P75 | P90 | P97 |
| --- | --- | --- | --- | --- | --- | --- | --- | --- | --- | --- | --- |
| 36 | 48.1 | 0.3 | 0.2 | 1.3 | 28.1 | 34.8 | 41.5 | 48.1 | 55.4 | 64.8 | 77.4 |
| 37 | 46.9 | 0.3 | 0.2 | 1.3 | 27.8 | 34.3 | 40.6 | 46.9 | 53.9 | 62.8 | 74.6 |
| 38 | 45.8 | 0.2 | 0.2 | 1.3 | 27.5 | 33.7 | 39.8 | 45.8 | 52.4 | 60.9 | 72.1 |
| 39 | 44.7 | 0.2 | 0.2 | 1.3 | 27.1 | 33.1 | 39.0 | 44.7 | 51.1 | 59.1 | 69.8 |
| 40 | 43.7 | 0.2 | 0.2 | 1.3 | 26.7 | 32.5 | 38.2 | 43.7 | 49.9 | 57.6 | 67.9 |
| 41 | 42.8 | 0.2 | 0.2 | 1.3 | 26.2 | 31.9 | 37.4 | 42.8 | 48.8 | 56.3 | 66.4 |
| 42 | 41.9 | 0.2 | 0.2 | 1.3 | 25.6 | 31.2 | 36.6 | 41.9 | 47.8 | 55.2 | 65.0 |
| 43 | 41.1 | 0.2 | 0.2 | 1.3 | 25.2 | 30.6 | 35.9 | 41.1 | 46.8 | 54.0 | 63.6 |
| 44 | 40.2 | 0.2 | 0.2 | 1.3 | 24.8 | 30.1 | 35.2 | 40.2 | 45.8 | 52.8 | 62.1 |
| 45 | 39.4 | 0.2 | 0.2 | 1.3 | 24.3 | 29.5 | 34.5 | 39.4 | 44.8 | 51.6 | 60.6 |
| 46 | 38.5 | 0.2 | 0.2 | 1.3 | 23.9 | 28.9 | 33.8 | 38.5 | 43.8 | 50.4 | 59.1 |
| 47 | 37.7 | 0.2 | 0.2 | 1.3 | 23.5 | 28.4 | 33.1 | 37.7 | 42.8 | 49.1 | 57.6 |
| 48 | 36.8 | 0.2 | 0.2 | 1.3 | 23.1 | 27.8 | 32.4 | 36.8 | 41.7 | 47.9 | 56.1 |
| 49 | 36.0 | 0.2 | 0.2 | 1.3 | 22.6 | 27.2 | 31.7 | 36.0 | 40.8 | 46.8 | 54.7 |
| 50 | 35.3 | 0.2 | 0.2 | 1.3 | 22.1 | 26.6 | 31.0 | 35.3 | 40.0 | 45.8 | 53.6 |
| 51 | 34.6 | 0.2 | 0.2 | 1.3 | 21.6 | 26.1 | 30.4 | 34.6 | 39.2 | 45.0 | 52.7 |
| 52 | 33.9 | 0.2 | 0.2 | 1.3 | 21.2 | 25.6 | 29.8 | 33.9 | 38.5 | 44.2 | 51.7 |
| 53 | 33.3 | 0.2 | 0.2 | 1.3 | 20.9 | 25.2 | 29.3 | 33.3 | 37.8 | 43.3 | 50.7 |
| 54 | 32.8 | 0.2 | 0.2 | 1.3 | 20.7 | 24.9 | 28.9 | 32.8 | 37.1 | 42.4 | 49.5 |
| 55 | 32.3 | 0.2 | 0.2 | 1.3 | 20.7 | 24.7 | 28.6 | 32.3 | 36.4 | 41.6 | 48.3 |
| 56 | 32.0 | 0.2 | 0.2 | 1.3 | 20.7 | 24.6 | 28.4 | 32.0 | 36.0 | 40.9 | 47.4 |
| 57 | 31.8 | 0.2 | 0.2 | 1.3 | 20.8 | 24.6 | 28.2 | 31.8 | 35.7 | 40.5 | 46.8 |
| 58 | 31.7 | 0.2 | 0.2 | 1.3 | 20.8 | 24.6 | 28.2 | 31.7 | 35.5 | 40.3 | 46.5 |
| 59 | 31.7 | 0.2 | 0.2 | 1.3 | 20.8 | 24.6 | 28.2 | 31.7 | 35.4 | 40.2 | 46.3 |
| 60 | 31.6 | 0.2 | 0.2 | 1.3 | 20.9 | 24.7 | 28.2 | 31.6 | 35.4 | 40.1 | 46.1 |

**S7. Percentile values for Pegboard – both hands in boys**

| AGE_M | mu | sigma | nu | tau | P3 | P10 | P25 | P50 | P75 | P90 | P97 |
| --- | --- | --- | --- | --- | --- | --- | --- | --- | --- | --- | --- |
| 36 | 112.0 | 0.2 | 0.1 | 1.5 | 75.2 | 87.0 | 99.0 | 112.0 | 126.5 | 143.4 | 164.4 |
| 37 | 109.4 | 0.2 | 0.1 | 1.5 | 73.1 | 84.7 | 96.6 | 109.4 | 123.8 | 140.6 | 161.5 |
| 38 | 106.9 | 0.2 | 0.1 | 1.5 | 71.0 | 82.4 | 94.2 | 106.9 | 121.1 | 137.7 | 158.5 |
| 39 | 104.4 | 0.2 | 0.1 | 1.5 | 69.1 | 80.3 | 91.9 | 104.4 | 118.5 | 134.9 | 155.4 |
| 40 | 102.0 | 0.2 | 0.1 | 1.5 | 67.3 | 78.3 | 89.7 | 102.0 | 115.9 | 132.1 | 152.4 |
| 41 | 99.8 | 0.2 | 0.1 | 1.5 | 65.5 | 76.4 | 87.6 | 99.8 | 113.5 | 129.5 | 149.5 |
| 42 | 97.6 | 0.2 | 0.1 | 1.5 | 63.9 | 74.6 | 85.6 | 97.6 | 111.2 | 127.0 | 146.8 |
| 43 | 95.7 | 0.2 | 0.1 | 1.5 | 62.3 | 72.9 | 83.8 | 95.7 | 109.1 | 124.7 | 144.4 |
| 44 | 93.9 | 0.2 | 0.1 | 1.5 | 61.0 | 71.4 | 82.2 | 93.9 | 107.1 | 122.6 | 142.0 |
| 45 | 92.1 | 0.2 | 0.1 | 1.5 | 59.9 | 70.1 | 80.6 | 92.1 | 105.1 | 120.3 | 139.4 |
| 46 | 90.4 | 0.2 | 0.1 | 1.5 | 58.9 | 68.9 | 79.2 | 90.4 | 103.0 | 117.8 | 136.3 |
| 47 | 88.5 | 0.2 | 0.1 | 1.5 | 58.0 | 67.7 | 77.7 | 88.5 | 100.7 | 115.0 | 132.9 |
| 48 | 86.6 | 0.2 | 0.1 | 1.5 | 57.0 | 66.4 | 76.1 | 86.6 | 98.4 | 112.2 | 129.4 |
| 49 | 84.7 | 0.2 | 0.1 | 1.5 | 56.0 | 65.2 | 74.5 | 84.7 | 96.1 | 109.4 | 126.0 |
| 50 | 82.9 | 0.2 | 0.1 | 1.5 | 55.1 | 64.0 | 73.0 | 82.9 | 93.9 | 106.7 | 122.7 |
| 51 | 81.0 | 0.2 | 0.1 | 1.5 | 54.3 | 62.8 | 71.6 | 81.0 | 91.6 | 103.9 | 119.3 |
| 52 | 79.3 | 0.2 | 0.1 | 1.5 | 53.5 | 61.8 | 70.2 | 79.3 | 89.5 | 101.3 | 115.9 |
| 53 | 77.8 | 0.2 | 0.1 | 1.5 | 52.8 | 60.8 | 69.0 | 77.8 | 87.6 | 98.9 | 112.9 |
| 54 | 76.4 | 0.2 | 0.1 | 1.5 | 52.2 | 60.0 | 67.9 | 76.4 | 85.9 | 96.8 | 110.4 |
| 55 | 75.2 | 0.2 | 0.1 | 1.5 | 51.6 | 59.2 | 66.9 | 75.2 | 84.3 | 94.9 | 108.0 |
| 56 | 74.0 | 0.2 | 0.1 | 1.5 | 51.1 | 58.5 | 66.0 | 74.0 | 82.9 | 93.2 | 105.8 |
| 57 | 72.9 | 0.2 | 0.1 | 1.5 | 50.5 | 57.7 | 65.1 | 72.9 | 81.6 | 91.6 | 104.0 |
| 58 | 72.0 | 0.2 | 0.1 | 1.5 | 50.0 | 57.1 | 64.3 | 72.0 | 80.5 | 90.3 | 102.4 |
| 59 | 71.1 | 0.2 | 0.1 | 1.5 | 49.5 | 56.5 | 63.6 | 71.1 | 79.4 | 89.0 | 100.8 |
| 60 | 70.3 | 0.2 | 0.1 | 1.5 | 49.2 | 56.0 | 62.9 | 70.3 | 78.4 | 87.8 | 99.3 |

**S8. Percentile values for Pegboard – both hands in girls**

| AGE_M | mu | sigma | nu | tau | P3 | P10 | P25 | P50 | P75 | P90 | P97 |
| --- | --- | --- | --- | --- | --- | --- | --- | --- | --- | --- | --- |
| 36 | 106.5 | 0.2 | 0.1 | 1.4 | 67.5 | 80.2 | 93.0 | 106.5 | 121.7 | 140.1 | 164.0 |
| 37 | 103.7 | 0.2 | 0.1 | 1.4 | 66.7 | 78.8 | 91.0 | 103.7 | 118.0 | 135.3 | 157.7 |
| 38 | 101.0 | 0.2 | 0.1 | 1.4 | 65.7 | 77.3 | 88.9 | 101.0 | 114.5 | 130.8 | 151.8 |
| 39 | 98.3 | 0.2 | 0.1 | 1.4 | 64.5 | 75.6 | 86.7 | 98.3 | 111.3 | 126.8 | 146.8 |
| 40 | 95.8 | 0.2 | 0.1 | 1.4 | 63.1 | 73.8 | 84.6 | 95.8 | 108.4 | 123.5 | 142.8 |
| 41 | 93.5 | 0.2 | 0.1 | 1.4 | 61.3 | 71.9 | 82.5 | 93.5 | 105.8 | 120.7 | 139.8 |
| 42 | 91.3 | 0.2 | 0.1 | 1.4 | 59.5 | 69.9 | 80.4 | 91.3 | 103.6 | 118.3 | 137.3 |
| 43 | 89.3 | 0.2 | 0.1 | 1.4 | 57.8 | 68.1 | 78.4 | 89.3 | 101.5 | 116.2 | 135.2 |
| 44 | 87.4 | 0.2 | 0.1 | 1.4 | 56.2 | 66.4 | 76.7 | 87.4 | 99.5 | 114.1 | 133.0 |
| 45 | 85.7 | 0.2 | 0.1 | 1.4 | 54.9 | 65.0 | 75.1 | 85.7 | 97.6 | 112.0 | 130.7 |
| 46 | 84.0 | 0.2 | 0.1 | 1.4 | 53.9 | 63.7 | 73.6 | 84.0 | 95.6 | 109.7 | 127.9 |
| 47 | 82.3 | 0.2 | 0.1 | 1.4 | 53.2 | 62.7 | 72.2 | 82.3 | 93.5 | 107.1 | 124.6 |
| 48 | 80.5 | 0.2 | 0.1 | 1.4 | 52.4 | 61.6 | 70.8 | 80.5 | 91.3 | 104.3 | 121.0 |
| 49 | 78.7 | 0.2 | 0.1 | 1.4 | 51.7 | 60.5 | 69.4 | 78.7 | 89.1 | 101.5 | 117.5 |
| 50 | 77.0 | 0.2 | 0.1 | 1.4 | 50.9 | 59.5 | 68.1 | 77.0 | 87.0 | 99.0 | 114.3 |
| 51 | 75.4 | 0.2 | 0.1 | 1.4 | 50.1 | 58.4 | 66.8 | 75.4 | 85.1 | 96.7 | 111.5 |
| 52 | 73.9 | 0.2 | 0.1 | 1.4 | 49.3 | 57.4 | 65.5 | 73.9 | 83.3 | 94.5 | 108.9 |
| 53 | 72.6 | 0.2 | 0.1 | 1.4 | 48.6 | 56.5 | 64.4 | 72.6 | 81.6 | 92.5 | 106.4 |
| 54 | 71.4 | 0.2 | 0.1 | 1.4 | 48.1 | 55.8 | 63.4 | 71.4 | 80.2 | 90.7 | 104.1 |
| 55 | 70.5 | 0.2 | 0.1 | 1.4 | 47.7 | 55.2 | 62.7 | 70.5 | 79.0 | 89.2 | 102.2 |
| 56 | 69.8 | 0.2 | 0.1 | 1.4 | 47.4 | 54.8 | 62.2 | 69.8 | 78.3 | 88.4 | 101.2 |
| 57 | 69.6 | 0.2 | 0.1 | 1.4 | 47.1 | 54.5 | 61.9 | 69.6 | 78.0 | 88.1 | 101.0 |
| 58 | 69.6 | 0.2 | 0.1 | 1.4 | 46.7 | 54.3 | 61.8 | 69.6 | 78.2 | 88.6 | 101.7 |
| 59 | 69.8 | 0.2 | 0.1 | 1.4 | 46.4 | 54.1 | 61.8 | 69.8 | 78.7 | 89.4 | 103.0 |
| 60 | 70.1 | 0.2 | 0.1 | 1.4 | 46.0 | 53.9 | 61.8 | 70.1 | 79.2 | 90.3 | 104.5 |
